# Pompe Disease: Pathogenesis, Molecular Mechanisms, Neurological Aspects, Diagnostics and Modern Therapeutic Approaches

**DOI:** 10.3390/ijms27083703

**Published:** 2026-04-21

**Authors:** Alexandra Sharshakova, Alisa Fattakhova, Valeriya Solovyeva, Albert Sufianov, Galina Sufianova, Grigorii Kutovoi, Albert Rizvanov

**Affiliations:** 1 Institute for Fundamental Medicine and Biology, Kazan Federal University, 420008 Kazan, Russia; alsharshakova@kpfu.ru (A.S.); alisashajmardanova@kpfu.ru (A.F.); vavsoloveva@kpfu.ru (V.S.); g2007risho@gmail.com (G.K.); 2 Department of Neurosurgery, Sechenov First Moscow State Medical University of the Ministry of Health of the Russian Federation (Sechenov University), 119991 Moscow, Russia; sufianov_a_a@staff.sechenov.ru; 3 The Research and Educational Institute of Neurosurgery, Peoples’ Friendship University of Russia (RUDN), 117198 Moscow, Russia; 4 Department of Pharmacology, Tyumen State Medical University, 625023 Tyumen, Russia; sufarm@mail.ru; 5 Division of Medical and Biological Sciences, Tatarstan Academy of Sciences, 420111 Kazan, Russia

**Keywords:** Pompe disease, CRIM status, lysosomal storage disease, neurodegeneration, autophagy, enzyme replacement therapy, gene therapy

## Abstract

Pompe disease (PD) is a neuromuscular autosomal recessive disorder caused by mutation in the *GAA* gene, which encodes acid α-glucosidase (GAA), an enzyme responsible for hydrolyzing glycogen to glucose. Deficiency of this enzyme leads to pathological accumulation of glycogen in almost all tissues of the body, with the most pronounced effects in cardiac and skeletal muscle, as well as in the central nervous system. Two major clinical forms of PD are recognized: infantile-onset PD, characterized by almost complete absence of GAA activity and severe cardiomyopathy and neurological abnormalities, and late-onset PD, which primarily presents with impairment of respiratory and motor function. Since 2006, enzyme replacement therapy with recombinant GAA has been used to treat PD, improving survival and quality of life. However, this approach has several limitations: the need for lifelong infusions, the risk of immune responses, and the inability of the enzyme to cross the blood–brain barrier, which is particularly critical for infantile-onset PD. Consequently, alternative strategies are being developed, including gene therapy using adeno-associated virus vectors for GAA delivery to target tissues; these approaches are currently in phase I/II clinical trials. Transplantation of genetically modified hematopoietic stem cells also represents a promising therapeutic strategy, offering a single-intervention treatment with long-lasting effects. This review discusses the molecular mechanisms of PD, current and emerging disease models, and therapeutic approaches, which together open prospects for the development of potentially one-time curative treatments, despite persistent challenges such as immunogenicity and the need for long-term efficacy monitoring.

## 1. Introduction

Pompe disease (PD), also known as glycogen storage disease type II (OMIM #232300), is a rare autosomal recessive neuromuscular disorder caused by deficiency of the lysosomal enzyme acid α-glucosidase (GAA) resulting from mutation in the *GAA* gene [1]. GAA is a soluble lysosomal enzyme that catalyzes the hydrolysis of glycogen to glucose in the acidic environment of the lysosome [2,3]. Impaired GAA function leads to glycogen accumulation in multiple tissues; however, skeletal, respiratory, and cardiac muscles are most severely affected [3].

PD occurs in approximately 1 in 40,000 newborns, although the estimated incidence ranges from 1 in 14,000 to 1 in 600,000 depending on ethnic and geographic factors. PD is most frequently reported among African American populations, with an estimated incidence of approximately 1 in 14,000 individuals, whereas in Portugal the disease is markedly rarer, with approximately one case diagnosed per 600,000 newborns [4]. These differences are likely due to population-specific genetic background, including varying frequencies of pathogenic *GAA* variants and pseudodeficiency alleles, as well as differences in diagnostic practices and newborn screening protocols across regions [4,5]. After newborn screening for PD was implemented in several U.S. states, the observed incidence rate increased, reaching approximately 1 in 8657 newborns [2,3]. A large-scale analysis of the Genome Aggregation Database provided a more granular picture of population-specific predicted genetic prevalence (pGP) across seven major population groups, revealing that the true global pGP of PD (1:23,232) substantially exceeds the conventionally cited estimate of 1:40,000. The highest pGP was observed in East Asian individuals (1:12,125). In contrast, Latino Americans, South Asians, and Finnish individuals exhibited considerably lower predicted prevalence. Importantly, East Asian and African populations harbored higher proportions of pathogenic variants associated with classic infantile-onset PD (IOPD). These findings highlight that current prevalence estimates may be systematically underestimated in populations with limited access to screening programs [4].

Currently, PD treatment is based on enzyme replacement therapy (ERT) with recombinant GAA (rhGAA), which was approved by the U.S. Food and Drug Administration (FDA) in 2006 [6]. Early results demonstrated improvements in patient survival and clinical outcomes [6,7]. However, this approach has several limitations, including residual muscle pathology, limited penetration across the blood–brain barrier (BBB), resulting in persistent central nervous system involvement, and immune responses against the infused enzyme [2,8]. In addition, ERT does not fully resolve extensive autophagic accumulation in muscle fibers, which further contributes to reduced therapeutic efficacy. These limitations, discussed in more detail below, highlight the need for the development of alternative therapeutic strategies, including gene therapy using adeno-associated viruses (AAVs) and lentiviral vectors (LVs) to enable long-term GAA expression [9,10]. Additional approaches include substrate reduction therapy (SRT), as well as combinations of ERT with pharmacological chaperones or gene therapy [2].

This review is a narrative, non-systematic overview of current approaches to the treatment of PD. The literature search was conducted using Google Scholar and PubMed databases. Articles were identified using combinations of the following keywords: “Pompe disease,” “glycogen storage disease type II,” “enzyme replacement therapy,” “gene therapy,” “AAV,” and related terms. In addition, information on ongoing and completed clinical studies was retrieved from the ClinicalTrials.gov database. The search primarily focused on peer-reviewed articles published within approximately the last 5–10 years, with earlier seminal studies included where relevant. Studies were selected based on their relevance to the pathophysiology of PD and current therapeutic strategies. No formal systematic review protocol was applied; however, articles were selected based on relevance, recency, and impact in the field.

The aim of this review is to summarize current knowledge on the pathogenesis and clinical manifestations of PD, as well as available and emerging disease models and therapeutic approaches.

## 2. Overview of PD

### 2.1. Clinical Forms of PD and Their Symptoms

The severity and rate of progression of PD depend on the age of clinical onset and the extent of residual GAA activity. Two main forms are recognized: IOPD, the most severe variant characterized by the near-complete absence of functional enzyme activity (GAA activity < 1%), and late-onset PD (LOPD), in which residual GAA activity may reach up to 30% (Table 1) [2,11]. Infants with IOPD are diagnosed within the first few months of life and, if untreated, die before the age of one year due to cardiac or respiratory failure [12]. Clinical manifestations include hypotonia, progressive muscle weakness, respiratory insufficiency, macroglossia, hepatomegaly, and hypertrophic cardiomyopathy [3,13]. IOPD is further classified into classic IOPD, characterized by severe cardiomyopathy and death within the first two years of life, and non-classic IOPD, which presents with a less severe form of cardiomyopathy and slowly progressive myopathy [11]. Symptoms of LOPD patients manifest after 12 months of age and predominantly involve motor and respiratory dysfunction, which may progress to loss of ambulation and the need for ventilatory support [3]. In contrast to IOPD, marked cardiomyopathy is typically absent in LOPD [11]. LOPD exhibits marked phenotypic heterogeneity, ranging from mild myopathic involvement to severe respiratory insufficiency, depending on age at onset and disease progression. In addition, the term LOPD also includes asymptomatic individuals identified through newborn screening programs or family history [14,15].

LOPD is subdivided into juvenile-onset PD (JOPD; onset at 1–18 years) and adult-onset PD (AOPD), with JOPD being the least characterized in terms of clinical presentation and genetic features [16]. Retrospective analyses indicate that JOPD manifests with a broad clinical spectrum, ranging from asymptomatic individuals identified by an elevated creatine kinase (CK) level to children with delayed motor development and, less commonly, gastrointestinal symptoms. In a subset of patients with JOPD, cardiomyopathy and respiratory involvement may be observed, partially resembling the clinical features of IOPD. However, in contrast to classic IOPD, the majority of JOPD patients harbor at least one “mild” *GAA* mutant allele, such as c.-32-13T>G, which preserves residual enzyme activity. In PD overall, a general genotype–phenotype trend is observed, where biallelic severe “null” variants (e.g., c.525delT) are typically associated with IOPD, while the presence of at least one mild allele is linked to later-onset forms [17]. Although the c.-32-13T>G variant—the most prevalent mutation in LOPD and JOPD—is associated with residual GAA activity, it exhibits considerable phenotypic variability. As a result, no specific genotypic feature reliably predicts clinical outcomes, including cardiac or respiratory involvement and the rate of motor decline [16]. This highlights the weak genotype–phenotype correlation in JOPD and underscores the limited predictive value of genotype alone for disease severity and progression [14,16]. Current knowledge of JOPD is largely derived from small retrospective cohorts and case series, resulting in an incomplete understanding of disease natural history, prognostic markers, and long-term therapeutic outcomes, thereby emphasizing the need for dedicated longitudinal studies specifically focused on the juvenile population.

**Table 1:** Summary table of clinical forms of Pompe disease.

Clinical Form		Disease Onset	Symptoms	Genetics	References
IOPD		First months of life	Hypotonia, progressive muscle weakness, respiratory insufficiency, macroglossia, hepatomegaly, hypertrophic cardiomyopathy, lesions of the white or gray matter, vascular abnormalities, developmental delay, hearing loss	Most frequent “Dutch mutations”: c.525delT (nucleotide deletion) and c.2481+102_2646+31del (exon 18 deletion), leading to the formation of a nonfunctional GAA protein	[3,13,18,19,20,21]
LOPD	JOPD	1–18 years	Muscle weakness, delayed motor development, respiratory insufficiency, skeletal changes	The most common variant is the splicing mutation c.-32-13T>G, which results in residual GAA activity	[22,23]
	AOPD	From 18 years of age	Motor function impairment, respiratory failure, seizures, strokes, hemorrhages, hearing loss	The most common variant is the splicing mutation c.-32-13T>G, which results in residual GAA activity	[3,18,24]

Recent clinical data from newborn screening programs across 22 U.S. states indicate that IOPD accounts for approximately 12% of diagnosed cases in the United States, whereas in Asian populations—predominantly in Taiwan—this proportion reaches approximately 28%, among whom 25% lack cross-reactive immunologic material (CRIM) [25,26]. In an analysis of 217 IOPD patients, 68 (31%) were CRIM-negative [27]. CRIM-negative individuals harbor two pathogenic *GAA* variants that result in complete loss of GAA protein production; consequently, these patients mount robust immune responses against ERT, complicating treatment. Overall, CRIM-negative status is associated with earlier disease onset, more aggressive clinical progression, and poorer prognosis [22].

#### 2.1.1. CRIM Status

A key determinant of ERT efficacy is the patient’s CRIM status, which reflects the presence or absence of endogenous GAA protein [28,29]. As noted previously, CRIM-negative patients completely lack native GAA due to pathogenic mutations. Clinical data demonstrate dramatic differences in treatment outcomes: during the first year of therapy, mortality reaches 54.5% among CRIM-negative patients, compared with 4.8% in the CRIM-positive group. CRIM-negative individuals also exhibit cardiac and motor function impairment, along with a poor prognosis for ventilator-free survival. In addition, these patients develop a more rapid immune response to recombinant enzyme and display higher anti-GAA antibody titers than CRIM-positive patients. Another retrospective study with children affected by PD showed that even CRIM-positive patients with high anti-GAA antibody titers had unfavorable outcomes across all clinical parameters, with no statistically significant differences from CRIM-negative patients [30]. Although most CRIM-positive patients develop low or intermediate anti-rhGAA antibody titers, a subset may also mount a clinically significant humoral response. The determinants of this variability are not fully understood, but available data suggest contributions from the underlying *GAA* genotype and other individual immunologic factors [29].

Current clinical guidelines recommend determining CRIM status prior to initiation of ERT, and this has become standard practice in the management of PD, particularly in IOPD patients, as CRIM-negative patients may develop treatment resistance to rhGAA, requiring immunomodulatory therapy [31]. Various agents and their combinations are being investigated to prevent the development of neutralizing antibodies against rhGAA by modulating immune responses and promoting tolerance to ERT. Immune tolerance induction (ITI) can involve combinations of rituximab, methotrexate, and intravenous immunoglobulin, with or without bortezomib (which induces apoptosis of antibody-producing cells and modulates immune responses), administered concomitantly with ERT [28,32,33]. Rituximab is a chimeric anti-CD20 antibody that depletes B cells and reduces autoimmune reactivity, whereas methotrexate is a folate antagonist that suppresses proliferation and activity of inflammatory cells [34,35].

#### 2.1.2. CNS Involvement in Patients with PD

PD is considered a disease that initially affects skeletal and cardiac muscles; however, there are accumulating data on involvement of the central nervous system (CNS) in the pathological process [18]. CNS involvement in PD has most often been reported in patients with IOPD: lesions of both white and gray matter, vascular abnormalities, cognitive impairments, and hearing loss were observed [18,19]. Increasing evidence from animal studies and from patients with PD suggests that deficiency of the GAA enzyme leads to pathological glycogen accumulation in neurons and glial cells [36,37,38]. Such forms of the disease do not respond to standard ERT, since the recombinant enzyme cannot cross the BBB [39].

Previously, neuroimaging results showed white matter changes in 18-month-old patients with IOPD [11,40]. Autopsy studies of PD patients revealed glycogen aggregates in brainstem and spinal motor neurons, in Schwann cells, and in oligodendrocytes [41,42]. In *GAA*-knockout mice, glycogen accumulation was found in neurons as well as in glial cells of the cortex, cerebellum, brainstem, and spinal cord. Motor neurons contained vacuoles filled with glycogen [42,43,44]. Torri et al. analyzed 81 studies describing CNS involvement in PD. About half of the works (48%) included 271 patients with IOPD, roughly 36% included 265 patients with LOPD, and the remainder focused on both forms. It was noted that in children with IOPD, demyelinating (leukoencephalopathic) lesions predominantly form in the white matter of the brain and cognitive development delays are observed. Among patients with LOPD, seizures, strokes, hemorrhages, and hearing loss were reported [18].

### 2.2. Genetic Basis of PD

The *GAA* gene is located on chromosome 17q25.2–q25.3 and contains 20 exons. The first exon does not code for a protein, while the remaining 19 exons encode an enzyme of 952 amino acids with a molecular weight of 105 kDa. Currently, more than 500 mutations in the *GAA* gene are known to cause PD. The genetic spectrum of mutations in *GAA* is highly diverse: many are characteristic for specific families or small populations [24]. Among possible sequence changes are (i) point mutations affecting the stability and function of the protein or disrupting splicing and (ii) deletions and insertions of varying sizes that can lead to the formation of unstable mRNAs. This, in turn, affects protein synthesis, its post-translational modifications, transport to lysosomes, and subsequent proteolytic processing [45].

**Figure 1:**
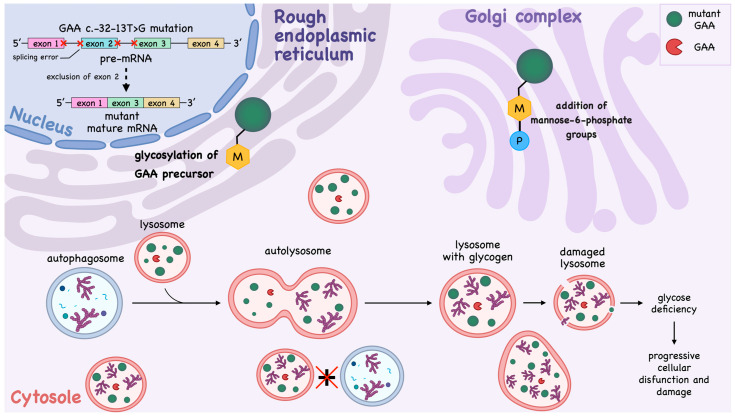
The pathogenesis of PD at the cellular level: mutations in the *GAA* gene result in the production of defective α-glucosidase enzyme, leading to impaired lysosomal glycogen degradation. As a consequence, glycogen is delivered to lysosomes via autophagy but cannot be efficiently metabolized, resulting in progressive lysosomal glycogen accumulation and reduced glucose availability. Lysosomal overload subsequently disrupts autophagic flux and normal cellular metabolism, ultimately causing cellular dysfunction and damage.

The most common nucleotide variant among individuals of European descent leading to childhood or adult-onset disease is the splice site mutation c.-32-13T>G (splice site variant, c.-32-13T>G in intron 1, IVS1). This intronic mutation affects the splicing of *GAA* mRNA, resulting in partial skipping of exon 2. As a result, a mixture of different mRNA isoforms is produced. Most transcripts are nonfunctional because the missing exon 2 disrupts protein coding. However, a small amount of correctly spliced transcript persists, leading to GAA synthesis (Figure 1). Thus, patients with this nucleotide variant inherit LOPD with residual GAA activity [20,46,47].

The most severe nucleotide variants in the *GAA* gene include c.525delT (deletion of a single nucleotide) and c.2481+102_2646+31del (deletion of exon 18), commonly referred to as “Dutch mutations” due to their high frequency in the Dutch population. Both mutations are severe because they cause a frameshift and a premature stop codon, rendering the encoded protein nonfunctional. Such nucleotide variants are most often found in patients with IOPD [20,21]. Genetic analysis of 243 IOPD patients showed that 25.1% had CRIM-negative status. Most of them had either homozygous or compound heterozygous nonsense mutations or frameshift mutations leading to premature stop codons or deletions of several exons [28,29]. CRIM-positive patients typically have missense mutations or in-frame deletions, in which at least a small amount of GAA is synthesized. Missense mutations are therefore generally associated with CRIM-positive status. However, in rare cases, certain point substitutions—such as variants affecting the initiator methionine codon (e.g., c.1A>G; p.Met1?) or missense variants that disrupt splicing—may result in a CRIM-negative phenotype [28,48,49].

It is noted that individuals with homozygous carriage of the pseudodeficiency allele show a pronounced decrease in GAA enzymatic activity, yet clinical manifestations of PD are absent in them [20,50]. Two genetic variants—c.1726G>A (p.Gly576Ser) and c.2065G>A (p.Glu689Lys)—commonly occur in cis configuration on the same allele and are associated with GAA pseudodeficiency. The first variant (c.1726G>A) markedly reduces both the level of GAA protein expression and its catalytic activity [20,51].

## 3. Molecular Biology of GAA: Biosynthesis, Processing and Transport

GAA is a ubiquitously expressed lysosomal enzyme present from early developmental stages; however, its expression levels vary markedly across tissues and cell types. In adult organisms, higher expression is observed in metabolically active tissues, including the nervous system, heart, liver, and skeletal muscle, whereas lower levels are detected in lung and immune-related tissues [52].

The biosynthesis of the GAA enzyme begins with a 110 kDa precursor in the rough endoplasmic reticulum (ER), where it undergoes several stages of modifications before reaching the lysosomes [53]. In the ER, precursor molecules undergo glycosylation, specifically the addition of oligosaccharides enriched with mannose residues. These carbohydrate modifications are necessary for proper protein folding and subsequent transport to the Golgi complex [54]. In the Golgi complex, the GAA protein undergoes phosphorylation of mannose residues on N-glycosylated sites, resulting in the addition of mannose-6-phosphate (Figure 1). This modification is crucial as it enables recognition of the protein by mannose-6-phosphate receptors (M6PRs), which mediate targeted transport of the enzyme to lysosomes [55].

The enzyme captured by M6PRs is packaged into vesicles budding from the Golgi and transported to endosomes. In the endosomes, the enzyme is released from the receptor complex and then transported to the lysosomes. Meanwhile, M6PRs recycle back to the Golgi for reuse, with a portion trafficked to the plasma membrane [3]. During transport to the lysosome, the enzyme undergoes proteolytic processing affecting both the amino and carboxyl termini, which is essential for its activation. The 110 kDa precursor is cleaved at the amino terminus to form a 95 kDa intermediate starting from amino acid 122; then, this 95 kDa intermediate is further processed at the carboxyl terminus into a 76 kDa form. At the final stage, the 76 kDa enzyme undergoes additional cleavage at the amino terminus and removal of N-linked oligosaccharide residues, becoming the mature 70 kDa form with full catalytic activity [20,56].

Under normal conditions, a small amount of the GAA precursor is secreted into the extracellular space. This extracellular precursor can be taken up by cells via the cation-independent mannose-6-phosphate receptor (CI-M6PR) on their surface and delivered to lysosomes, where in acidic environment it participates in glycogen degradation [55]. This mechanism forms the basis of enzymatic cross-correction in PD, which is utilized in ERT and *GAA* gene therapy to ensure delivery of active enzyme to target tissues and cells [2].

## 4. Autophagy and PD

As previously described, in PD, undegraded glycogen accumulates in multiple tissues, with a predominance in muscular tissues (both skeletal and cardiac). PD represents one of the first pathological conditions associated with autophagy, also referred to as “self-eating” [24]. Autophagy is an evolutionarily conserved catabolic process whose primary function is the lysosomal degradation of intracellular components [24,57]. It plays a crucial role in cellular energy homeostasis, as well as in the turnover and renewal of macromolecules in skeletal muscle. Impaired autophagy in muscle tissue results in mitochondrial damage, ER stress, defective turnover of sarcomeric proteins, and ultimately, cell death [57,58].

The autophagic process involves the formation of double-membrane vesicles known as autophagosomes, which capture cytoplasmic components. Following sequestration, autophagosomes fuse with lysosomes to form autophagolysosomes, where the enclosed material is degraded by lysosomal enzymes [59]. In recent years, autophagy has been shown to be a selective process capable of specifically targeting certain cellular components in response to distinct physiological cues [60]. Among the forms of selective autophagy, glycophagy—responsible for the degradation of cellular glycogen within autophagic vacuoles—plays a key role in maintaining glucose homeostasis. The captured glycogen is degraded by GAA, leading to the release of α-glucose, which is rapidly available for cellular energy production [24,61].

### 4.1. Molecular Mechanisms Underlying Autophagy and Their Dysregulation in PD

The initiation of autophagy in mammalian cells is a complex, tightly regulated process in which the unc-51 like autophagy activating kinase 1 (ULK1) serves as a key upstream regulator of the autophagic cascade [62]. The activity of ULK1 is precisely controlled by two opposing signaling systems: the inhibitory mammalian target of rapamycin complex 1 (mTORC1) and the activating adenosine monophosphate-activated protein kinase (AMPK). AMPK stimulates autophagy by phosphorylating ULK1 at Ser317 and Ser777 residues, whereas mTORC1 inhibits autophagy initiation by phosphorylating ULK1 at alternative sites, thereby preventing AMPK-ULK1 interaction [57,63,64]. Under nutrient-deprived conditions, inhibition of mTORC1 activity allows AMPK to initiate autophagic signaling, providing the cell with alternative energy sources [57]. These findings indicate that AMPK-induced autophagy degrades intracellular proteins to supply nutrients and energy under metabolic stress [65].

The influence of AMPK and mTORC1 on muscle pathophysiology has been investigated in GAA-deficient multinucleated myotubes and skeletal muscle tissues of *GAA*-knockout mice. Studies revealed reduced mTORC1 activity, evidenced by decreased phosphorylation of its downstream targets—4E-binding protein 1 (4E-BP1) and ribosomal protein S6 kinase 1 (S6K1)—and the subsequent reduction in phosphorylation of ribosomal protein S6 [63]. Concurrently, activation of AMPK was observed, accompanied by phosphorylation of its downstream effectors, tuberous sclerosis complex 2 (TSC2) and acetyl-CoA carboxylase (ACC). These molecular alterations coincide with an elevated ADP/ATP ratio, indicative of an energy deficit, which further promotes AMPK-dependent autophagy activation [63,66,67].

The principal mechanism contributing to muscle pathology in PD is a block in autophagic flux—specifically, the impairment of autophagosome-lysosome fusion (Figure 1). Confocal microscopy of cultured muscle fibers derived from *GAA*-knockout mice expressing red fluorescent protein-tagged LAMP1 (a lysosomal marker, Lysosome-Associated Membrane Protein 1) and green fluorescent protein-tagged LC3 (a specific autophagosomal marker, Microtubule-Associated Protein 1A/1B-Light Chain 3) revealed a critical reduction in lysosome number and an almost complete absence of autophagosome-lysosome fusion events [54,63,68].

To address the defects resulting from autophagic blockade, the transcription factor EB (TFEB) has been investigated as a potential therapeutic target [63]. TFEB coordinates and regulates the expression of numerous genes involved in the autophagic process, including genes responsible for autophagy initiation, substrate capture, autophagosome trafficking, and fusion with lysosomes [59,69]. Experimental overexpression of TFEB in PD mouse models led to near-complete clearance of accumulated autophagic material. Interestingly, a similar effect was observed upon activation of mTORC1 through genetic inhibition of TSC2, which restored protein synthesis and reversed muscle atrophy [63,68,70]. Thus, TFEB may represent a complementary therapeutic strategy in combination with ERT or gene therapy; however, the available evidence is currently limited to preclinical studies.

Oxidative stress in PD is largely driven by impaired autophagy, which leads to the accumulation of dysfunctional mitochondria and increased reactive oxygen species (ROS) production. Studies in cellular and murine models of PD have identified multiple mitochondrial abnormalities, including dysregulated Ca^2+^ homeostasis and increased ROS production. In one preclinical study, pharmacological blockade of Ca^2+^ channels partially reversed these abnormalities, suggesting that modulation of calcium handling may represent a potential adjunctive therapeutic strategy; however, this approach remains at the stage of limited preclinical investigation and requires further validation [71]. In addition, oxidative stress has emerged as another therapeutically relevant secondary mechanism in PD. Antioxidants such as N-acetylcysteine, idebenone, resveratrol, and edaravone were shown to enhance rhGAA efficacy in patient-derived cells and in the PD mouse model by improving enzyme processing and trafficking, supporting their potential use as adjunctive therapies [72].

### 4.2. Autophagy in PD Patients and Its Impact on Therapeutic Efficacy

Autophagic abnormalities in PD were first documented in 1970, when Andrew Engel described unusual inclusions in muscle biopsies from adult patients [73]. At that time, the molecular mechanisms of autophagy were poorly understood, and its role in disease pathogenesis remained unrecognized for decades [54].

Autophagy has since been extensively studied both in mouse models and in patients with PD, with particular attention given to the effects of ERT on autophagic inclusions. Autophagic processes in PD were investigated using Myozyme (the first-generation ERT) in *GAA*-knockout mice [63]. These studies demonstrated that treatment successfully corrected cardiac pathology and normalized glycogen levels in cardiac muscle. In contrast, therapeutic outcomes in skeletal muscle were considerably less effective: uptake of recombinant enzyme in skeletal muscle was markedly lower than in cardiac or hepatic tissues, and glycogen clearance occurred only partially, particularly in fast-twitch glycolytic type II fibers, where glycogen content remained largely unchanged [74].

Recent findings have identified a key mechanism underlying the limited efficacy of ERT in skeletal muscles of PD patients. The impaired delivery of recombinant enzyme to lysosomes is caused by extensive autophagic accumulation, which obstructs enzyme trafficking. This phenomenon reflects the close functional interplay between the endocytic and autophagic pathways, which converge into a unified lysosomal degradation system [75]. Experimental data have shown that, under pathological conditions, rhGAA predominantly accumulates within central autophagic regions of type II muscle fibers—those most resistant to ERT—whereas under normal physiological conditions, the enzyme is efficiently trafficked to lysosomes [76,77].

Muscle biopsies from PD patients have revealed pathological patterns similar to those observed in *GAA*-knockout mice. A characteristic feature of LOPD samples is the heterogeneity of muscle fiber involvement, reflecting the progressive nature of the disorder. In mildly affected regions, LAMP2- and LC3-positive vesicles were observed, indicating defective degradation of autophagosomes within lysosomes. In other areas, a distinct “autophagic core” was present, consisting of aggregated autophagosomes and lysosomes localized in the central region of muscle fibers, disrupting the contractile apparatus. In the most severely affected fibers, a complete disorganization of myofibrillar structure and loss of CI-M6PR expression were detected [75,78].

Collectively, these findings indicate that the highest therapeutic efficacy of standard ERT is achieved in patients with minimal pre-existing muscle damage. Clinical observations have confirmed that individuals with lower baseline levels of muscular pathology exhibit better responses to therapy [75,79].

The limited efficacy of Myozyme in skeletal muscles prompted the development of next-generation ERT formulations. A key limitation of the traditional preparation was its low content of mannose-6-phosphate (M6P), a critical ligand required for binding to the CI-M6PR, which mediates lysosomal targeting of the enzyme. Only a small fraction of the administered rhGAA possessed high receptor affinity, substantially reducing treatment efficiency [63,80]. The characteristics and constraints of next-generation ERT will be discussed later in this review in the Section 7.

## 5. Diagnosis of PD

The diagnosis of PD is often challenging due to its heterogeneous symptomatic manifestation. Many symptoms overlap with those of other myopathies and neuromuscular disorders, particularly in LOPD [19]. As a result, diagnostic delays are common, with physicians often identifying PD only after excluding more prevalent pathologies. The diagnostic delay varies significantly depending on the age of symptom onset: approximately 1.4 months for newborns presenting with cardiomyopathy, and up to six years for patients whose first symptoms appear after the age of 12. Patients with symptom onset within the first 12 months of life or between 1 and 12 years of age tend to experience the longest diagnostic delays [24].

The laboratory diagnosis of PD is based on the detection of reduced GAA enzyme activity combined with confirmation of two pathogenic variants in the *GAA* gene [81,82]. The most convenient and rapid screening method—especially in newborns—is the measurement of GAA activity in dried blood spots (DBSs). However, no unified standard exists for result documentation, and positive DBS results require confirmation through more specific assays [82,83]. For confirmatory testing, GAA activity may be measured in leukocytes, cultured skin fibroblasts, or muscle tissue. Most laboratories utilize the fluorometric substrate 4-methylumbelliferyl-α-D-glucopyranoside for GAA activity assays. To exclude the activity of other α-glucosidases, such as maltase-glucoamylase, the inhibitor acarbose is typically added [81,82].

Prenatal diagnosis of PD is based on molecular genetic and/or enzymatic testing of fetal samples. When familial *GAA* pathogenic variants are known, targeted genetic analysis can be performed using DNA obtained from chorionic villus sampling or amniocentesis. Alternatively, GAA enzyme activity can be measured in chorionic villi or cultured amniocytes to confirm the diagnosis [19,84].

In addition, patients with PD may exhibit elevated serum levels of CK, aspartate aminotransferase, alanine aminotransferase, and lactate dehydrogenase. However, these biochemical markers are not specific and may remain within normal ranges in some individuals with PD [3,24].

Measurement of the urinary tetrasaccharide 6-α-D-glucopyranosyl-maltotetraose (Glc4) is also used as a supportive diagnostic tool for both IOPD and LOPD patients [82]. Glc4 is a product of glycogen degradation by GAA, which is secreted into plasma and subsequently excreted in urine [85]. Although Glc4 is normally present at low levels, its concentration increases under pathological conditions associated with glycogen accumulation, such as PD [86]. However, this biomarker is nonspecific, as elevated urinary Glc4 levels can also occur in glycogen storage diseases type III and VI, Duchenne muscular dystrophy, and hepatic dysfunction [82,86]. Urinary Glc4 is sometimes assessed together with its structural isomer, maltotetraose (M4), which is also generated during glycogen degradation. Although M4 can be elevated in PD, it has not been widely adopted in diagnostic practice due to its limited stability in urine and associated analytical challenges. While this limitation may be partially mitigated by optimizing sample pH conditions, Glc4 remains the clinically validated and most widely used biomarker, whereas M4 has not been incorporated into routine diagnostics [87].

Electromyography may support a presumptive diagnosis of PD. In paraspinal muscles, spontaneous activity (fibrillations and positive sharp waves) and short-duration, early-recruited motor unit potentials are often observed [28]. Muscle biopsy can provide additional diagnostic information in muscle diseases. In patients with IOPD, characteristic histopathological findings include PAS-positive material (Periodic acid–Schiff staining of glycogen), cytoplasmic vacuoles, and acid phosphatase activity—indicative of glycogen accumulation in muscle fibers [15,31]. However, PAS-positive vacuolar myopathy is not specific to PD and may yield false negative results. Therefore, biopsy findings must always be corroborated by enzymatic or genetic tests. Because muscle biopsy is an invasive procedure, non-invasive methods such as DBS testing are preferred for initial screening [31].

Molecular genetic testing aims to identify pathogenic mutations in the *GAA* gene [24]. Sanger sequencing remains the most commonly used method; however, multiplex ligation-dependent probe amplification is applied to detect large deletions or duplications. When conventional approaches provide insufficient resolution, advanced molecular techniques such as next-generation sequencing, minigene analysis, single-nucleotide polymorphism genotyping, and splicing defect assays may be employed [24,28]. In cases of suspected disease, it is also important to consider the possibility of pseudodeficiency states caused by certain combinations of *GAA* gene variants [82]. In Asian populations, variants that reduce GAA activity to borderline pathological values are common, which can yield false positive results in some tests [88]. In some cases, mutations may be missed by standard sequencing, warranting extended molecular-genetic analysis [82].

As discussed previously, determination of CRIM status is critical for PD patient management. Several methods are available, including Western blot analysis using cultured skin fibroblasts; however, this procedure is invasive and requires several weeks for results. A faster and less invasive alternative involves Western blot analysis of peripheral blood mononuclear cells, allowing for CRIM status determination within two to three days. This approach represents a practical alternative to fibroblast-based assays. However, its application may be limited by technical factors, including protein degradation, strict sample handling requirements, and limited cell yield, particularly in young patients [31,89,90]. In cases where pathogenic variants are already known, CRIM status can be predicted based solely on molecular analysis of the *GAA* gene in approximately 92% of patients [48]. The main limitation in the remaining cases is the presence of variants—particularly splice site changes—whose effect on GAA protein synthesis cannot be reliably inferred from DNA sequence alone [29].

Integrated digital platforms that combine artificial intelligence (AI), clinical data, genetic information, and data from wearable devices are being developed to optimize PD diagnosis, monitoring, and personalized care. AI-based tools have been applied for automated phenotyping and rare disease identification using electronic health records [91,92], while wearable devices have been used to monitor mobility, respiratory parameters, and physical activity in patients with late-onset PD [93]. In addition, mobile applications enabling remote monitoring and patient self-management are emerging as supportive tools in clinical care [94]. Such digital health solutions represent an important advancement toward improving clinical management and enabling data-driven research in neuromuscular disorders [95].

## 6. PD Models

### 6.1. In Vitro PD Models

In vitro models are used to study the pathophysiology and evaluate the therapeutic efficacy in PD, including myoblast lines—some generated using CRISPR/Cas9 technology and carrying specific *GAA* gene mutations—as well as induced pluripotent stem cells (iPSCs) derived from PD patients [24,96,97]. Cellular models of PD have been developed based on myoblasts isolated from *GAA*-knockout mice and differentiated into myotubes, as well as from satellite cells of single muscle fibers. These models demonstrated a characteristic PD phenotype, including enlarged, alkalinized lysosomes filled with glycogen. However, they did not exhibit the key autophagic pathology. In addition, these cellular models were difficult to culture and had limited proliferative potential [98,99]. Studies of PD and ERT response using myoblasts derived from patients with LOPD revealed mitochondrial dysfunction and an energy crisis within the cells. Nevertheless, this cellular model failed to fully reproduce the autophagic pathology and showed substantial variability in the ERT response between patient-derived cell lines [100].

Several studies have also established immortalized cell lines from *GAA*-knockout mice. These cells are capable of unlimited proliferation, allowing for multiple passages, unlike primary myoblasts. However, such cell models reproduce only lysosomal abnormalities and do not reflect the autophagic defects characteristic of PD [54,99,101].

Another limitation of using primary myoblasts or immortalized cell lines from animal models of PD is that they do not carry the same mutations found in PD patients. To investigate mutation-specific disease mechanisms and develop personalized therapeutic approaches, CRISPR/Cas9 genome editing has been employed [96,99]. Kan et al. introduced a mutation characteristic of patients with IOPD into mouse myoblasts using CRISPR/Cas9. This cellular model exhibited glycogen accumulation, impaired autophagy, and nearly absent GAA activity. Subsequently, transgenic mice carrying the same mutation were generated and displayed a PD phenotype with significant glycogen accumulation, muscle weakness, and cardiomyopathy. However, these mice did not exhibit neonatal lethality, which is typical of IOPD patients [96]. In another study, CRISPR/Cas9 genome editing was used to generate mouse myoblasts carrying severe *GAA* mutations. These cells showed complete loss of GAA activity, elevated glycogen levels, impaired autophagy, and reduced expression of CI-M6PR [99].

Patient-derived iPSCs from individuals with PD can be differentiated into skeletal muscle cells, hepatocytes, cardiomyocytes, and neuronal stem cells [102]. Yoshida et al. systematically developed iPSC-based models of PD that recapitulate the key cellular phenotypes of the disease across different tissues. In their initial study, the authors differentiated iPSCs from patients with IOPD into myocytes and demonstrated that these cells were characterized by lysosomal glycogen accumulation, impaired energy metabolism, and mitochondrial dysfunction. Subsequently, the same group reported the generation of hepatocytes from iPSCs derived from IOPD patients, which also accumulated significant amounts of glycogen. In both cases, treatment with rhGAA resulted in a dose-dependent reduction in glycogen levels, confirming the suitability of these models for studying disease mechanisms and for drug screening [103,104]. Several studies have also generated cardiomyocytes from iPSCs of PD patients. These models reproduced the major pathological hallmarks of the disease—lysosomal glycogen accumulation, mitochondrial dysfunction, and a pronounced response to ERT. However, no significant autophagic abnormalities were detected, likely due to culture-specific conditions and the fact that autophagic defects are more characteristic of later disease stages [97,102,105]. As previously mentioned, particularly IOPD symptom manifestation is associated with neurodegenerative changes. Consequently, several studies have focused on developing nervous system models, including terminally differentiated neurons and neuronal stem cells derived from iPSCs of IOPD patients. These cells exhibited glycogen accumulation and reduced GAA activity, making them useful for evaluating potential therapeutic compounds. Thus, Cheng et al. demonstrated that hydroxypropyl-β-cyclodextrin (a cyclic oligosaccharide) and δ-tocopherol (a component of vitamin E) act synergistically to enhance the efficacy of ERT in neuronal stem cells, as evidenced by a reduction in autophagic activity. These two structurally unrelated small molecules had previously been identified as agents capable of reducing lysosomal storage in several lysosomal storage disorders (LSD), primarily by stimulating lysosomal exocytosis [102,106,107].

To investigate therapeutic responses, the first three-dimensional skeletal muscle model derived from myoblasts of IOPD patients was developed. This model reproduced key features of PD, including reduced GAA activity, enlarged lysosomes, glycogen accumulation, and impaired contractile function. Application of ERT and transduction with an AAV vector carrying a functional *GAA* gene led to decreased glycogen levels and restoration of GAA activity; however, contractile dysfunction of the muscle tissue persisted [108].

### 6.2. In Vivo PD Models

Animal models carrying genetic defects associated with PD have been identified in several species, including cattle, dogs, cats, sheep, and Japanese quail [54,109]. Notably, Finnish and Swedish Lapphunds exhibit a frameshift mutation analogous to that found in patients with IOPD [110]. These animals develop pathological changes in skeletal and smooth muscles, the heart, and motor neurons. Byrne et al. investigated systemic and intrathecal administration of AAV vectors carrying the human *GAA* gene. This gene therapy approach prevented the development of cardiomyopathy, improved skeletal muscle function, and increased GAA activity in both muscle tissue and neurons, thereby demonstrating the translational relevance of these animal models for developing therapeutic strategies for PD [111]. Japanese quail display a milder form of the disease without cardiac involvement, likely due to the expression of alternative α-glucosidase isoforms that provide residual GAA activity [54]. However, these species are less practical for PD research compared with conventional transgenic laboratory animals because of their substantial evolutionary distance from humans (e.g., quail) and low reproductive rates (e.g., cattle) [109].

To study PD in vivo, several mouse lines with targeted *GAA* gene disruption have been developed. The first model was generated by deleting exon 13, resulting in a complete loss of GAA activity and glycogen accumulation in the heart, liver, and skeletal muscles. These mice recapitulated the IOPD phenotype. A second model, created by replacing exon 6 with a neomycin resistance cassette, displayed features consistent with both IOPD and LOPD [54,109,112,113].

A transgenic mouse line expressing a GFP reporter fused to the autophagosome marker LC3 has also been described. These mice demonstrated key autophagic pathology along muscle fibers and mitochondrial dysfunction. Screening of AAV-based gene therapy delivering human *GAA* in this model revealed restoration of mitochondrial function and a reduction in autophagic structures, which could be visualized in vivo in real time using intravital microscopy [114].

Bragato et al. generated a zebrafish (*Danio rerio*) model of PD to investigate disease pathophysiology and facilitate drug screening using 3-bromopyruvate. These models reproduced hallmark features of PD, including excessive glycogen accumulation, motor defects, and impaired autophagy. 3-Bromopyruvate, an inhibitor of hexokinase—a key glycolytic enzyme—has known antitumor activity in phenotypes reliant on glycolysis for adenosine triphosphate production and proliferation. When tested in the zebrafish PD model, the 3-bromopyruvate reduced glycogen accumulation and improved motor performance [115].

Although GAA is highly conserved across species, with about 80% amino acid identity between human and mouse enzymes, important differences in sequence and processing exist. The catalytic core, including key residues such as Asp518 and Asp616, of the enzyme is strictly conserved, supporting cross-species modeling of functionally critical variants [116]. Pathogenic variants associated with IOPD, particularly those leading to severe loss of enzyme activity, are most reliably recapitulated in animal models [96]. In contrast, variants associated with LOPD—especially splicing defects such as c.-32-13T>G—are more difficult to reproduce due to low conservation of non-coding sequences and species-specific differences in post-translational processing [117].

## 7. Therapeutic Approaches for PD

### 7.1. Enzyme Replacement Therapy

#### 7.1.1. Historical Background

A fundamental basis for the development of ERT for LSDs was the discovery of the mechanism of enzyme uptake via the M6PR. Subsequent studies elucidated the critical role of the CI-M6PR on the cell surface, through which secreted lysosomal enzymes enter the endocytic pathway and ultimately reach lysosomes. This natural process of “cross-correction” became the conceptual foundation for the development of ERT, including therapy for PD [3,24,118,119]. The first in vitro ERT experiments were conducted in cultured skeletal muscle cells using GAA purified from bovine testis and human urine. These experiments demonstrated that GAA was internalized by lysosomes through M6PR or insulin-like growth factor receptors, resulting in the degradation of accumulated glycogen [120,121]. However, animal studies revealed that most of the administered enzyme accumulated in the liver and spleen, while only minimal amounts reached the heart and skeletal muscles. These findings predicted the need for high enzyme doses—on the order of tens of milligrams per kilogram of body weight—to achieve therapeutic efficacy in primary target tissues [121]. Similar conclusions were reached in in vivo experiments conducted in quail and *GAA*-knockout mice [74,121,122].

The situation changed with the successful cloning of the *GAA* gene, which enabled the development of recombinant enzyme production systems. Two production platforms were established: expression of rhGAA in the milk of transgenic rabbits and production in Chinese hamster ovary (CHO) cell cultures [121]. The milk-derived enzyme was produced using the full *GAA* gene sequence under the control of the αS1-casein promoter, whereas CHO cells were engineered using a complementary DNA (cDNA) construct of the *GAA* gene [123,124].

In 1999, the first clinical trials of rhGAA derived from the milk of transgenic rabbits were initiated in patients with PD. Six infants with classic IOPD, two adolescents, and one adult received treatment for 3–5 years before transitioning to the CHO-derived enzyme [125,126]. In parallel, studies of rhGAA produced in CHO cells were initiated, including trials in three infants [127]. Ultimately, the CHO-derived enzyme became the basis for large-scale industrial production [121].

#### 7.1.2. Alglucosidase Alfa (Myozyme/Lumizyme)

In 2006, rhGAA produced in CHO cells was approved by the FDA and registered under the names Myozyme™ in Europe and Lumizyme™ in the United States (Table 2). This was the first approved ERT for the treatment of both IOPD and LOPD [121,128]. Alglucosidase alfa (ALGLU) also became the first recombinant enzyme officially approved for treating a disease in which skeletal muscle is the primary target tissue [3]. ALGLU is administered intravenously every two weeks at a standard dose of 20 mg per kilogram of body weight [2]. In infants with IOPD whose symptoms manifested within the first six months of life, ALGLU markedly reduced cardiac hypertrophy, improved cardiac function, enhanced motor function, and significantly decreased the risk of death and the need for invasive ventilation [79]. However, with longer follow-up—up to 6 years—an increasing number of patients became ventilator-dependent, and some lost previously acquired motor skills [129].

Clinical studies have demonstrated substantial benefits of implementing newborn screening followed by early initiation of ERT in IOPD. Additionally, the potential for improving clinical outcomes through higher or more frequent ALGLU dosing has been investigated [31,141,142]. Gelder et al. conducted a study involving eight infants with IOPD: one group received ERT every two weeks at 20 mg/kg, while the other received weekly infusions at 40 mg/kg. The results showed 100% survival in both groups. Seven of eight patients maintained independent respiration, and significant improvements in motor function were observed in the higher-dose group. No major differences were detected between the groups in terms of left-ventricular mass reduction or antibody formation [143].

Nevertheless, treatment with ALGLU faces several limitations, particularly in patients with IOPD. The immune response against rhGAA represents a major challenge, especially the development of high and sustained antibody titers [2]. Such titers occur more commonly in CRIM-negative patients, who lack endogenous CRIM, although approximately 30% of CRIM-positive patients may also develop high titers [144]. Antibodies against the infused enzyme can compromise clinical efficacy through several mechanisms: (i) blocking catalytic activity; (ii) accelerating the clearance of rhGAA from circulation, thereby reducing delivery to muscle; (iii) interfering with lysosomal uptake by blocking receptor binding; or (iv) a combination of these mechanisms [80,145]. Clinical observations have shown that antibody titers can decrease following ITI, and in some cases high titers fail to develop when ITI is initiated before the start of ERT [19,32,146].

Another limitation of ALGLU is the inability of the recombinant enzyme to cross the BBB, rendering ERT ineffective against neurological manifestations [39,42]. In a cohort study, Hsu et al. examined CNS disease progression in CRIM-positive IOPD patients receiving ERT. Brain magnetic resonance imaging revealed progressive white matter abnormalities extending into U-fibers, the cerebellum, brainstem, and gray matter, despite early ERT. The extent of CNS involvement positively correlated with age and negatively correlated with IQ. Cerebral atrophy was observed in some patients [38]. Kenney-Jung et al. also analyzed IOPD patients treated with ALGLU and reported seizures, encephalopathy, marked white matter abnormalities, and stagnation of cognitive development beyond a certain age [147].

ERT in LOPD has demonstrated meaningful but variable outcomes. During the first three years of therapy, patients frequently exhibited improvements in motor function, as reflected by the six-minute walk test (6MWT), along with stabilization of respiratory function [130]. However, long-term therapeutic effects remain limited. A substantial proportion of patients (35–63%) exhibited diminished clinical response after 3–5 years of treatment [148]. A plateau effect after the initial improvement was also frequently observed [3]. Unlike in IOPD, the development of antibodies against the recombinant enzyme is not a major concern in LOPD. However, patients with LOPD may experience hypersensitivity reactions to intravenous infusions. Preventive measures include premedication with antihistamines and corticosteroids, adjustment of infusion rates, and gradual dose escalation to the therapeutic level [149].

In real-world clinical practice, ERT with ALGLU demonstrates initial improvements in functional parameters, including the 6MWT, muscle strength, and respiratory function. However, many patients experience a plateau or gradual decline in clinical benefit after several years of treatment, particularly over long-term follow-up (~7–10 years) [150]. Real-world evidence further indicates that, despite ERT, patients with PD continue to experience a substantial disease burden, characterized by high rates of comorbidities, reliance on supportive care, and persistent functional limitations [151]. Although ERT is an approved standard of care for PD, significant disparities in access to therapy persist across countries. High treatment costs, insurance limitations, variability in governmental reimbursement policies, and the need for specialized infusion infrastructure result in unequal opportunities for patients in both developed and developing healthcare systems to initiate and maintain ERT. In some African countries, access to ALGLU is extremely limited or utterly unavailable, leading to prolonged delays in treatment initiation and progressive disease deterioration [152].

#### 7.1.3. Second-Generation ERT (Avalglucosidase Alfa)

The limited efficacy of ALGLU in skeletal muscle, discussed in Section 4, led to the development of second-generation ERT. A key limitation of ALGLU is its insufficient content of M6P residues—particularly bis-phosphorylated oligosaccharides—which impairs binding of rhGAA to CI-M6PR and consequently restricts lysosomal delivery of the enzyme [63,128]. In response to these limitations, avalglucosidase alfa (Nexviazyme™/Nexviadyme™, AVA) was approved in 2021 for infusion therapy in patients with LOPD aged one year and older in the United States, and in Europe for both LOPD and IOPD [153] (Table 2).

Initial studies in mice demonstrated a fivefold improvement in glycogen clearance compared to the earlier rhGAA formulation [154]. The safety, pharmacokinetics, and pharmacodynamics of AVA were evaluated in an open-label phase 1 clinical trial and later confirmed in a six-year extension study [155]. In patients with LOPD transitioning from ALGLU to AVA, motor function improved, although respiratory outcomes ranged from minimal changes to deterioration [156]. In naïve LOPD patients, 49 weeks of AVA therapy improved forced vital capacity (FVC), motor function, and was found to be both safe and effective [131,157]. In a study of children with IOPD who showed disease progression or poor response to ALGLU, transitioning to AVA led to improved outcomes in most patients within 6 months. Higher-dose AVA (40 mg/kg) produced superior biomarker profiles compared with lower-dose AVA (20 mg/kg) or continued ALGLU therapy [132]. These findings indicate that AVA at 40 mg/kg may serve as an alternative for IOPD patients experiencing reduced efficacy or insufficient response to ALGLU-based ERT [133]. Additionally, AVA is currently under investigation as a treatment for infants (under 12 months) with previously untreated IOPD (NCT04910776). The primary outcome measures of this trial include overall survival and ventilator-free survival (NCT04910776).

Real-world studies suggest that switching from ALGLU to AVA may stabilize or improve motor and, in some cases, respiratory outcomes in patients with LOPD who exhibit plateauing or decline on long-term first-generation ERT, although responses remain heterogeneous [156,158]. Moreover, despite regulatory approval in multiple regions, access to AVA remains variable across healthcare systems and may be influenced by reimbursement policies and national funding frameworks.

#### 7.1.4. Second-Generation ERT (Cipaglucosidase Alfa-Atga+ Miglustat)

In 2023, another second-generation ERT was approved: cipaglucosidase alfa-atga (Pombiliti™) administered in combination with oral miglustat (Opfolda™) (Table 2) [134]. Cipaglucosidase alfa-atga is a rhGAA enriched with M6P residues to enhance lysosomal delivery, while miglustat is a small-molecule chaperone that stabilizes rhGAA in plasma [2,134]. Miglustat has been used for decades in other LSDs (e.g., Gaucher disease type 1, Niemann–Pick disease type C) as SRT through inhibition of glycosphingolipid synthesis. In the context of PD, miglustat mimics the terminal glucose of glycogen, which is the natural substrate of GAA, and, thus, binds competitively and reversibly to the active site of rhGAA, stabilizing the enzyme (cipaglucosidase alfa) in blood circulation [159]. Unlike AVA, in which additional M6P residues are introduced via chemical conjugation, the recombinant enzyme in Pombiliti™ is naturally expressed with a high content of bis-phosphorylated oligosaccharides [160]. The combination of cipaglucosidase alfa-atga and miglustat was approved following completion of a phase 3 clinical trial in adults with LOPD who showed insufficient clinical benefit from standard ERT. The trial compared the efficacy and safety of Pombiliti™ + Opfolda™ versus ALGLU in both ERT-experienced and ERT-naïve patients. Among previously treated patients, switching to the two-component therapy resulted in significant improvements in 6MWT distance and stabilization of FVC, whereas those continuing ALGLU did not demonstrate meaningful clinical progress. However, in ERT-naïve patients, Pombiliti™ + Opfolda™ and ALGLU produced comparable outcomes. In the pooled analysis, the new therapy did not demonstrate statistical superiority overall, and therefore it was approved only for patients who do not respond sufficiently to standard ERT [135]. In the ongoing open-label study, improvements in motor and respiratory function, as well as disease biomarker levels, were sustained up to week 104 regardless of prior ERT exposure [161]. A clinical trial is also underway (NCT04808505) evaluating intravenous cipaglucosidase alfa together with oral miglustat in patients with IOPD, including both ERT-naïve individuals and those previously treated with other ERT regimens (NCT04808505). The efficacy, safety, pharmacokinetics, pharmacodynamics, and immunogenicity of cipaglucosidase alfa in combination with miglustat are currently being evaluated in a phase III clinical trial involving pediatric patients (<18 years) diagnosed with late-onset Pompe disease (NCT03911505).

#### 7.1.5. Developing ERT Approaches

Several additional ERT strategies are being explored to improve enzyme delivery to skeletal muscle (Table 2). One such approach is reveglucosidase alfa—a chimeric protein composed of rhGAA fused to a fragment of human insulin-like growth factor 2 (IGF2). This fusion enhances cellular uptake of the enzyme through the CI-M6PR, also known as the insulin-like growth factor 2 receptor (IGF2R) [2,162]. In phase I/II clinical trials (NCT01230801, NCT01435772), reveglucosidase alfa was evaluated in patients with LOPD and showed therapeutic efficacy, but it induced hypoglycemia, likely due to activation of IGF2 signaling pathways [136].

VAL-1221 is another chimeric protein composed of a Fab-fragment of a cell-penetrating antibody, which uses the equilibrative nucleoside transporter ENT-2 to enter the cytosol, fused to rhGAA. As a result, VAL-1221 targets both lysosomal and cytosolic glycogen. This construct was tested in a phase I/II clinical trial in LOPD patients. VAL-1221 demonstrated good tolerability and safety, but further clinical evaluation was discontinued (NCT02898753) [137].

Other studies have investigated ERT combined with β2-agonists such as albuterol and clenbuterol. These agents may serve as potential adjunct therapies for PD because they modulate the structure, biochemistry, and function of skeletal muscle and are already used in muscular dystrophies [163]. β2-agonists induce smooth muscle relaxation and bronchodilation by increasing intracellular cyclic adenosine monophosphate and also possess immunomodulatory functions [156,157]. In a phase I/II study (NCT01885936) involving LOPD patients, the addition of albuterol to ERT demonstrated safety and potential therapeutic benefit. Participants showed improvements in respiratory and motor function, although effects on glycogen accumulation in muscle were limited [138]. Another phase I/II clinical trial (NCT01942590) evaluated clenbuterol in combination with ALGLU in LOPD patients. Eight participants exhibited improved motor function, confirmed by the 6MWT, and skeletal muscle biopsies showed enhanced glycogen clearance [139].

One of the most promising strategies for treating IOPD is in utero ERT, currently investigated in a phase I clinical trial for PD and eight other LSDs (NCT04532047). A severe family history—two children who died from IOPD with cardiomyopathy—enabled early diagnosis of the disease in the fetus. The fetus, diagnosed with CRIM-negative IOPD, received ALGLU therapy at a dose of 20 mg/kg estimated fetal weight via the umbilical vein every two weeks from 24 to 34 weeks of gestation. After birth, the infant continued receiving intravenous ALGLU (40 mg/kg) along with ITI using rituximab due to the presence of neutralizing antibodies against rhGAA. Clinical outcomes were remarkable: by 13 months of age, the child exhibited normal development with no typical IOPD-associated pathology, maintained normal cardiac function, showed stable growth parameters, and had normal biomarker levels [140].

Anti-TfR-GAA is a chimeric protein consisting of an antibody targeting the transferrin receptor (TfR) fused to rhGAA. This construct allows for transport of the enzyme across the BBB and its delivery to the CNS, as well as efficient uptake by cardiac and skeletal muscle. In murine models of PD, Anti-TfR-GAA reduced glycogen levels in the brain and spinal cord, decreased neuroinflammation, and showed comparable or superior efficacy to conventional ERT in the heart and skeletal muscle [164].

### 7.2. Gene Therapy Approaches

Innovative gene therapy approaches for the treatment of PD are currently under active development (Table 3). The most advanced strategies involve in vivo gene therapy using AAV vectors, which enable delivery of a therapeutic *GAA* expression cassette directly to target tissues, including skeletal muscle, the liver, and the CNS. Several of these platforms have already reached clinical trials and have demonstrated substantial therapeutic potential. In parallel, ex vivo approaches are being explored in preclinical studies, involving genetic modification of hematopoietic stem cells (HSCs) using LV followed by transplantation. These emerging strategies offer promising solutions to overcome the limitations of conventional ERT by enabling long-term enzyme expression, facilitating CNS delivery, and minimizing immune responses [165,166,167].

#### 7.2.1. In Vivo Gene Therapy

A key step in the development of gene therapy for PD is the selection of an optimal AAV serotype, promoter, and route of administration. In several studies, ubiquitous promoters—such as the human cytomegalovirus (CMV) promoter or the chicken β-actin promoter—have been used to achieve broad transgene expression in skeletal and cardiac muscle. Although these promoters ensure strong and sustained expression, they also increase the risk of immunogenicity [165]. Intravenous delivery of AAV vectors presents two major challenges: (i) effective transduction of skeletal muscle typically requires high vector doses (10^13^–10^14^ vg/kg), increasing the likelihood of immune responses; and (ii) targeted delivery to the CNS requires specific AAV serotypes capable of crossing the BBB. Local CNS administration routes (intrathecal, intraventricular, or cisterna magna injection) permit efficient transduction at lower doses but limit systemic enzyme distribution. The liver is also considered a “depot” for systemic secretion of GAA due to several advantages: high transduction efficiency at relatively low AAV doses and the capacity to promote immune tolerance to the transgene product [165,166,173].

A preclinical study evaluated an AAV vector carrying a codon-optimized human *GAA* cDNA (coGAA) under the control of a mouse muscle creatine kinase (MCK) enhancer/promoter combination (AAV8-eMCK-GAA, AT845) in PD mice models and in toxicology studies in non-human primates (NHPs). Systemic administration of the vector in mice increased GAA activity and promoted glycogen clearance in skeletal and cardiac muscle. In NHPs, intravenous vector delivery also increased GAA activity in the muscle and heart. However, high doses of human GAA induced xenogeneic immune responses in NHPs, which were not observed when macaque GAA was used. NHPs also exhibited degeneration of dorsal root ganglion (DRG) neurons [174]. DRG toxicity has been described in multiple preclinical studies of systemic and intrathecal delivery of high doses of various AAV serotypes, including AAV8 and AAV9. Histopathological lesions including sensory neuron degeneration, chromatolysis, and secondary axonopathy have been reported in NHPs across different serotypes, and appear to correlate with high systemic doses rather than a particular capsid, promoter, or transgene—suggesting a dose-dependent class effect [175,176]. An open phase I/II clinical trial (NCT04174105) is currently evaluating the safety and efficacy of AT845 in CRIM-positive LOPD patients. Interim results presented at a conference showed a favorable safety profile over two years in the first four treated patients, with reversible adverse events. One patient receiving the high dose developed grade 2 peripheral sensory neuropathy. All patients demonstrated muscle transduction, and three were able to discontinue ERT while maintaining stable biomarker profiles [168]. Reports of DRG toxicity in human AAV clinical trials remain limited. Regulatory safety summaries from FDA-monitored programs indicate the occurrence of dorsal root ganglion histopathological findings in some intrathecal AAV trials, while associated clinical symptoms are generally mild or subclinical, underscoring the need for careful sensory and electrophysiological monitoring in high-dose AAV-based therapies [177].

AAV8-LSP-GAA (ACTUS-101), encoding wild-type *GAA* under the control of a liver-specific promoter (LSP), is being evaluated in a phase I/II clinical trial in LOPD patients (NCT03533673). ACTUS-101 features a unique regulatory cassette that restricts GAA expression to hepatocytes. This localized expression promotes regulatory T-cell induction and immune tolerance to GAA. The first report from low-dose systemic administration showed increased GAA activity in serum and muscle after 52 weeks, along with stable motor and respiratory function. However, no glycogen clearance in muscle was observed, likely due to the low vector dose [169].

Another phase I/II clinical trial (NCT04093349; SPK-3006) is ongoing in LOPD patients. This study uses a modified AAVrh74 capsid (Spk100), which encodes a GAA enzyme optimized for enhanced secretion and is targeted to the liver [178,179]. Results from this clinical trial have not yet been published. In preclinical studies, SPK-3006 increased GAA activity in cardiac and skeletal muscle, as well as in the spinal cord and brain, in PD mice and NHPs [179].

The AAV9-DES-GAA vector, carrying *coGAA* under the control of a desmin (DES) muscle-specific promoter, was designed for the treatment of all forms of PD. Preclinical studies in PD mice demonstrated that this vector corrected pathological abnormalities in skeletal and cardiac muscle, supporting progression to clinical testing. A phase I clinical trial (NCT02240407) is currently enrolling six LOPD patients to assess safety, biodistribution, and the possibility of AAV re-administration, using direct intramuscular injection. The protocol includes pre-injection immunomodulation (rituximab + sirolimus, an mTOR inhibitor with immunomodulatory and tolerogenic properties) and two intramuscular injections into the tibialis anterior muscle, four months apart. Efficacy assessments include evaluation of neutralizing antibodies against the viral capsid and GAA enzyme, as well as biochemical and muscle parameters. Results from this trial have not yet been published [180,181].

Two clinical trials have also been conducted in IOPD patients. In one trial (NCT00976352), AAV1-CMV-GAA—driven by the ubiquitous CMV promoter—was injected into the diaphragm of nine ventilator-dependent IOPD patients. The study reported no serious adverse events, improved respiratory function, and increased duration of spontaneous breathing one year after treatment. All patients continued ERT in parallel. Three CRIM-negative patients underwent ITI, which reduced antibody levels against both AAV1 and GAA [170,171].

A phase I/II clinical trial for IOPD patients younger than six months is currently underway (NCT05793307). The study involves intravenous administration of AAV9-GAA (GC301) under the control of a ubiquitous promoter (not specified), following two preparatory doses of standard ERT. A 58-day prednisone regimen was used to prevent immune reactions. After 10 weeks of follow-up, encouraging outcomes were reported: no serious adverse events, normalization of circulating GAA activity, improved cardiac and motor function, and absence of anti-GAA antibodies [172]. An additional trial is enrolling LOPD patients older than six years for GC301 gene therapy (NCT06391736).

Despite encouraging results from several clinical trials, the clinical translation of AAV-based gene therapy remains limited. A major constraint is the narrow therapeutic window: effective transduction of skeletal muscle and/or the CNS requires high vector doses, which are associated with dose-dependent toxicity, most notably DRG injury [182]. In addition, manufacturing challenges represent a substantial limitation. Scaling up vector production to doses required for the treatment of neuromuscular disorders in adult patients is associated with high costs, variability in the ratio of full to empty capsids, and an increased risk of undesirable immune responses related to this heterogeneity [183].

The choice of serotype and promoter is another critical determinant, as it significantly affects both efficacy and safety. Serotype defines tissue tropism: for example, AAV9 efficiently transduces skeletal muscle, cardiac tissue, and the CNS; however, high systemic doses have been associated with DRG toxicity. Strong ubiquitous promoters (e.g., CMV) enhance transgene expression but may increase the risk of immune activation and toxicity, whereas tissue-specific promoters (e.g., MCK, DES, LSP) reduce off-target expression [182,183]. Safety should be considered the result of the interplay between capsid, promoter, and dose, necessitating optimization of vector design to minimize toxic effects while maintaining sufficient therapeutic efficacy.

A substantial proportion of the population harbors pre-existing neutralizing antibodies against common AAV serotypes, which can significantly impair transduction efficiency and often exhibit cross-reactivity across capsids. Furthermore, the development of neutralizing antibodies following a single administration generally precludes effective re-dosing with the same serotype. Contemporary strategies to manage the immune response include immunomodulatory approaches aimed at inducing immune tolerance, switching serotypes or modifying the route of administration, and engineering novel capsids with reduced immunogenicity [184,185]. Therefore, in the development of gene therapy for PD, priority is given to maximizing the efficacy and durability of a single administration while minimizing vector dose and immunogenicity.

#### 7.2.2. Ex Vivo Gene Therapy

Gene therapy approaches involving allogeneic hematopoietic stem cell transplantation (HSCT) are widely used for the treatment of inherited metabolic disorders, including a number of neurometabolic diseases [186]. However, experience with allogeneic HSCT in PD is extremely limited. Only a single case of HSCT in an infant with IOPD has been reported, but long-term evaluation was not possible due to the patient’s development of pneumonia and septicemia [187]. More promising is autologous HSCT, which has already been approved for metachromatic leukodystrophy, X-linked adrenoleukodystrophy, and β-thalassemia [188,189,190]. Autologous HSCT-based therapies involve the collection of CD34^+^ cells from a patient’s peripheral blood, followed by transduction of these cells with a LV vector carrying a functional *GAA* gene. The genetically modified HSCs are then reinfused into the patient after conditioning to create bone marrow niches that allow for effective engraftment [191]. In experimental models, conditioning typically involves the alkylating agent busulfan or total body irradiation [192].

Although no clinical trials of HSCT-based therapy have yet been conducted for PD, preclinical studies have shown encouraging results. In PD mouse models, increases in GAA enzymatic activity and improvements in neurological function have been documented [193,194]. A research program led by Stok and colleagues has progressively developed an HSCT-based gene therapy strategy for PD using genetically modified HSCs. In the initial study, the authors used an LV vector carrying the wild-type *GAA* gene to modify mouse HSCs. While this approach achieved supraphysiological levels of GAA activity in hematopoietic cells and restored normal GAA activity in most target tissues, it had major limitations, including incomplete correction of skeletal muscle pathology and minimal impact on glycogen accumulation in the CNS [193]. In a subsequent study, the researchers used a third-generation LV vector delivering *coGAA*. This modified strategy demonstrated substantial improvements, including increased GAA activity in skeletal and cardiac muscle and in the CNS, better motor performance, and reduced glycogen storage in internal organs, heart, and skeletal muscles [194].

In vitro studies have shown that fusing GAA to an IGF2 fragment to create an IGF2-tagged chimeric protein enhances enzyme secretion by donor cells and its subsequent uptake by recipient cells in a transwell system. This effect is mediated by the IGF2 fragment’s ability to bind specifically to CI-M6PR, improving transport and internalization of the enzyme [195]. HSCT-based gene therapy using coGAA fused to an IGF2 tag has also been evaluated in PD mouse models, demonstrating improvements in cardiac and motor function, prevention of autophagic abnormalities, and reductions in glycogen accumulation in the brain [196]. Based on these findings, an LV vector expressing an IGF2-tagged GAA chimeric protein under control of the ubiquitous MND promoter (Myeloproliferative sarcoma virus Enhancer, Negative control region deleted, dl587rev primer binding site substituted promoter) was developed. The study evaluated nine chimeric GAA variants, including versions carrying an ApoE tag (apolipoprotein E) to enhance CNS delivery, and variants containing a modified IGF2 tag with an R37A mutation that prevents IGF2 degradation while preserving receptor-binding activity. This modification is known as GILT (Glycosylation-Independent Lysosomal Targeting), a technology that uses an IGF2 fragment to achieve high-affinity CI-M6PR binding and enhanced lysosomal uptake. Among the constructs tested, the MND-GILT-R37A-GAAco vector demonstrated superior glycogen reduction in skeletal muscle and improved CNS pathology compared with unmodified coGAA [191,197].

### 7.3. Alternative Therapeutic Approaches for PD

SRT is applied for the treatment of several LSDs. SRT is based on the use of small molecules that inhibit glycosphingolipid biosynthesis, thereby reducing the accumulation of substrate. A major advantage of such small molecules is their ability to cross the BBB, consequently, enabling the potential correction of neurological manifestations [198]. In PD, an SRT strategy targeting the reduction in glycogen accumulation via inhibition of glycogen synthase 1 (GYS1) has been explored (Table 4). In preclinical studies, the small-molecule GYS1 inhibitor MZE-101 demonstrated promising results both as monotherapy and in combination with ERT. In mouse models, it reduced glycogen accumulation in skeletal muscles and restored normal cellular morphology [199]. However, GYS1 inhibition requires careful monitoring: genetic data indicate that moderate reduction in muscle glycogen is tolerated without adverse effects, whereas GYS1 deficiency in the heart can lead to arrhythmias, cardiomyopathy, and sudden death [200,201]. MZE-101 has been evaluated in a phase I clinical trial (NCT05249621) in healthy volunteers to assess safety, pharmacokinetics, and pharmacodynamics. Oral administration of MZE-101 was well tolerated, did not adversely affect cardiac function, and reduced glycogen accumulation in skeletal muscles [202].

The IVS1 mutation, which causes skipping of exon 2, is the most common among PD patients. Therefore, antisense oligonucleotides (ASOs) based on small nuclear RNAs are being investigated to promote inclusion of exon 2 in mature mRNA and restore normal splicing [204]. The efficacy of this approach has been demonstrated in patient fibroblasts and multinucleated myotubes [128,204]. ASOs targeting GYS1 inhibition have also been explored. They have been delivered as naked oligonucleotides, conjugated to receptor-binding peptides, or via AAV vectors [2]. These approaches showed partial reduction in glycogen accumulation in muscles and synergistic effects with ERT [205]. Small interfering RNA conjugated with the Cetryrin protein and targeting transferrin receptor type 1 (TfR1) is currently in phase I clinical trials to evaluate its safety and tolerability (ABX1100, NCT06109948).

## 8. Conclusions

PD imposes a substantial and lifelong burden on patients and their families. Progressive muscle weakness, respiratory impairment, fatigue, and reduced mobility significantly affect quality of life, social participation, educational and professional opportunities, and psychological well-being.

To date, no effective therapy capable of completely curing PD exists. Advances in understanding the molecular mechanisms of the disorder, including autophagy impairment and CNS involvement in disease pathogenesis, have enabled the identification of new therapeutic targets. Emerging adjunctive approaches aimed at correcting secondary cellular defects are being explored, including modulation of autophagy (e.g., via TFEB activation) and strategies targeting mitochondrial dysfunction and oxidative stress. However, these approaches are currently supported predominantly by preclinical studies and require further validation before clinical translation.

ERT has significantly improved patient outcomes over recent years, particularly in LOPD. However, this approach is associated with several limitations, including the need for lifelong infusions, the development of immune responses in CRIM-negative patients, and the inability to correct neurological symptoms due to a lack of BBB penetration. In addition, patients frequently report a therapeutic plateau after several years of ERT, and in some countries ERT remains prohibitively expensive, with severely limited access. The caregiver burden remains substantial, particularly in infantile-onset and severe late-onset forms of PD that require respiratory or motor support. Therefore, future clinical trials should incorporate the assessment of patient-reported outcomes, health-related quality of life, and caregiver impact. Such measures will enable a comprehensive evaluation of emerging therapeutic strategies and help determine their true impact on patients’ daily lives.

In utero ERT represents a promising direction and has already shown encouraging results. Nevertheless, despite the progress achieved, this approach—similar to postnatal ERT—does not enable enzyme delivery across the BBB and therefore does not address CNS involvement. In addition, questions remain regarding the durability of therapeutic effects, necessitating continued long-term follow-up. Although immune responses can be partially controlled through ITI regimens combined with ERT, neurological manifestations and accumulation of autophagic vacuoles remain major obstacles to achieving a definitive cure for PD. Against this background, alternative therapeutic approaches are being actively developed.

Gene therapy for PD is currently undergoing intensive clinical investigation and already demonstrates promising results in both IOPD and LOPD. The use of different AAV serotypes, delivery routes, and tissue-specific promoters enables targeted transduction of skeletal and cardiac muscles and opens opportunities for improved gene delivery to the CNS. However, gene therapy may not fully address pathological processes associated with the presence of dysfunctional, misfolded, or truncated GAA variants, which may continue to exert deleterious effects at the cellular level.

HSC-based gene therapy is currently in preclinical development and has shown high efficacy in mouse models. This strategy enables a reduction in glycogen accumulation in cardiac and skeletal muscles as well as in the CNS. Leveraging stem cells provides the possibility of a one-time intervention capable of sustaining long-term GAA expression and, consequently, long-lasting therapeutic benefit.

Several additional therapeutic strategies—including SRT, ASOs, and modified ERT variants such as chimeric rhGAA proteins and combinations of ERT with β_2_-agonists—are currently in clinical trials and show potential for enhancing overall treatment efficacy.

Thus, continued investigation of PD pathogenesis, together with ongoing clinical evaluation of emerging therapeutic approaches, paves the way toward more effective and potentially one-time treatments capable of overcoming the limitations of current therapies. At present, in utero ERT represents one of the most conceptually promising strategies, as it offers the possibility of modifying disease progression at the earliest developmental stages, potentially before irreversible tissue damage occurs. However, clinical experience remains extremely limited, and larger cohorts with long-term follow-up will be required to determine the durability of benefit and safety. Among emerging modalities, AAV-mediated gene therapy is currently the most advanced in terms of clinical translation. Nevertheless, careful optimization of vector dose, serotype selection, promoter design, and route of administration remains essential to maximize therapeutic efficacy while minimizing immune responses and dose-dependent toxicity. Critical milestones to monitor include durable GAA expression, sustained glycogen clearance in skeletal and cardiac muscle, evidence of CNS benefit where applicable, and the feasibility of large-scale vector manufacturing.

Collectively, the near-term clinical landscape is likely to be shaped primarily by refinements in ERT and the maturation of AAV-based gene therapy programs. The future standard of care for PD will depend on integrating molecular efficacy, long-term safety, patient-reported outcomes, and real-world accessibility.

## Figures and Tables

**Table 2:** Enzyme replacement therapy strategies for Pompe disease.

Drug	Patients	Status/Clinical Trials ID	Advantages	Disadvantages	References
Alglucosidase alfa	IOPD and LOPD	Approved by FDA (Myozyme^TM^/Lumizyme^TM^)	Improvement in motor and respiratory function in LOPD patients (6MWT, FVC); marked reduction in hypertrophic cardiomyopathy in IOPD patients	Development of anti-drug antibodies; inability to cross the BBB; reduced long-term efficacy, particularly in skeletal muscle	[2,38,79,129,130]
Avalglucosidase alfa	IOPD and LOPD	Approved by FDA (Nexviazyme™/Nexviadyme™)	Greater improvement in patient-reported respiratory symptoms and improved skeletal muscle targeting compared to first-generation ERT in LOPD patients; stabilization or modest improvement of motor and cardiac functions in IOPD patients	Development of anti-drug antibodies; inability to cross the BBB	[131,132,133]
	naïve IOPD	Phase III/Active, not recruiting NCT04910776	Results not yet published	Results not yet published	
Cipaglucosidase alfa-atga in combination with oral miglustat	LOPD	Approved by FDA (Pombiliti™ + Opfolda™)	Clinically meaningful improvements and stabilization of motor and respiratory function, with durable effects observed in long-term studies	Did not achieve statistical superiority over first-generation ERT for the primary endpoint (change in 6MWD); development of anti-drug antibodies; inability to cross the BBB	[2,134,135]
	LOPD (≤18 y.o.)	Phase III/Active, not recruiting NCT03911505	Results not yet published	Results not yet published	
	IOPD	Phase III/Recruiting NCT04808505	Results not yet published	Results not yet published	
Reveglucosidase alfa	LOPD	Phase I/II/Completed NCT01230801 Phase II/Terminated NCT01435772	Improved respiratory muscle strength and walking endurance	High immunogenicity; frequent hypoglycemia; occurrence of serious adverse events	[136]
VAL-1221 chimeric protein	LOPD	Phase I/II/Terminated NCT02898753	Dose-dependent improvements in walking distance and muscle function	Infusion-associated reactions; immunogenicity	[137]
Alglucosidase alfa in combination with albuterol	LOPD	Phase I/II/Completed NCT01885936	Improvements in motor and respiratory functions	Limited impact on muscle glycogen clearance and lack of disease-modifying effect	[138]
Alglucosidase alfa in combination with clenbuterol	LOPD	Phase I/II/Completed NCT01942590	Improvements in motor and respiratory functions with reduced muscle glycogen content	Transient increase in CK level	[139]
In utero alglucosidase alfa	IOPD	Phase I/Recruiting NCT04532047	Cardiac function improvement, absence of typical IOPD symptoms	Limited clinical data	[140]

**Table 3:** Gene therapy strategies for Pompe disease.

Drug	Patients	Status/Clinical Trials ID	Advantages	Disadvantages	References
AAV8-eMCK-GAA (AT845)	LOPD	Phase I/II/Active, not recruiting NCT04174105	Muscle-specific *hGAA* expression with sustained biomarker stabilization and ERT withdrawal	Transient transaminitis; isolated serious adverse events	[168]
AAV8-LSP-GAA (ACTUS-101)	LOPD	Phase I/II/Active, not recruiting NCT03533673	Increased GAA activity in serum and muscles; stable motor and respiratory functions	Administration of a low dose resulted in limited glycogen clearance in muscles	[169]
AAVSk100-LSP-GAA (SPK-3006)	LOPD	Phase I/II/Active, not recruiting NCT04093349	Results not yet published	Results not yet published	
AAV9-DES-GAA	LOPD	Phase I/Completed NCT02240407	Results not yet published	Results not yet published	
AAV1-CMV-GAA	IOPD	Phase I/II/Completed NCT00976352	No immune response observed following ITI regimen	Risk of immune response without ITI; short-term follow-up	[170,171]
AAV9-GAA (GC301) under the control of a ubiquitous promoter	IOPD	Phase I/II/Active, not recruiting NCT05793307	Cardiac and motor functions improvement; sustained increase in plasma GAA activity to normal range	Persistent elevation of liver enzymes; short follow-up period	[172]
	LOPD	Phase I/II/Recruiting NCT06391736	Results not yet published	Results not yet published	

**Table 4:** Substrate reduction therapy strategies for Pompe disease.

Drug	Patients	Status/Clinical Trials ID	Advantages	Disadvantages	References
Small-molecule GYS1 inhibitor (MZE-101/MZE-001)	Healthy volunteers	Phase I/Completed NCT05249621	The drug demonstrated safety and good tolerability	Further monitoring is required	[199,200,201,202]
GYS1-targeting miRNA-Cetrytin (ABX1100)	Healthy volunteers	Phase I/Active, not recruiting NCT06109948	Preliminary data demonstrated decreases in CK and Glc4 levels.	Limited clinical data	[203]

## Data Availability

No new data were created or analyzed in this study. Data sharing is not applicable to this article.

## Abbreviations

The following abbreviations are used in this manuscript:

4E-BP1	4E-binding protein 1
6MWT	Six-minute walk test
AAV	Adeno-associated virus
ACC	Acetyl-CoA carboxylase
ALGLU	Alglucosidase alfa
AMPK	Adenosine monophosphate-activated protein kinase
AOPD	Adult-onset Pompe disease
ASOs	Antisense oligonucleotides
AVA	Avalglucosidase alfa
BBB	Blood–brain barrier
CHO	Chinese hamster ovary
CI-M6PR	Cation-independent mannose-6-phosphate receptor
CK	Creatine kinase
CMV	Cytomegalovirus
CNS	Central nervous system
coGAA	Codon-optimized acid α-glucosidase
CRIM	Cross-reactive immunologic material
DBS	Dried blood spots
DES	Desmin promoter
DRG	Dorsal root ganglion
ER	Endoplasmic reticulum
ERT	Enzyme-replacement therapy
FDA	U.S. Food and Drug Administration
GAA	Acid α-glucosidase
Glc4	Tetrasaccharide 6-α-D-glucopyranosyl-maltotetraose
GYS1	Glycogen synthase 1
HSCs	Hematopoietic stem cells
HSCT	Hematopoietic stem cell transplantation
IGF2	Insulin-like growth factor 2
IGF2R	Insulin-like growth factor 2 receptor
IOPD	Infantile-onset Pompe disease
iPSCs	Induced pluripotent stem cells
ITI	Immune tolerance induction
IVS1	Splice site variant, c.-32-13T>G in intron 1
JOPD	Juvenile-onset Pompe disease
LAMP1	Lysosome-associated membrane protein 1
LC3	Microtubule-associated protein 1a/1b-light chain 3
LOPD	Late-onset Pompe disease
LSD	Lysosomal storage disorder
LSP	Liver-specific promoter
LV	Lentivirus
M6P	Mannose-6-phosphate
M6PR	Mannose-6-phosphate receptor
MCK	Muscle creatine kinase
mTORC1	Mammalian target of rapamycin complex 1
NHPs	Non-human primates
PAS	Periodic acid–Schiff staining of glycogen
PD	Pompe disease
pGP	Predicted genetic prevalence
rhGAA	Recombinant acid α-glucosidase
ROS	Reactive oxygen species
S6K1	Protein S6 kinase 1
SRT	Substrate reduction therapy
TFEB	Transcription factor EB
TfR	Transferrin receptor
TfR1	Transferrin receptor type 1
TSC2	Tuberous sclerosis complex 2
ULK1	Unc-51 like autophagy activating kinase 1

## References

[1] Byrne B.J., Kishnani P.S., Case L.E., Merlini L., Müller-Felber W., Prasad S., van der Ploeg A. (2011). Pompe Disease: Design, Methodology, and Early Findings from the Pompe Registry. Mol. Genet. Metab..

[2] Colella P. (2024). Advances in Pompe Disease Treatment: From Enzyme Replacement to Gene Therapy. Mol. Diagn. Ther..

[3] Kohler L., Puertollano R., Raben N. (2018). Pompe Disease: From Basic Science to Therapy. Neurotherapeutics.

[4] Park K.S. (2021). Carrier Frequency and Predicted Genetic Prevalence of Pompe Disease Based on a General Population Database. Mol. Genet. Metab. Rep..

[5] Giugliani R., Solomon F., Kushlaf H., Wright E., Haselkorn T., Zanoteli E., Schoser B. (2025). Global Variations in Diagnostic Methods and Epidemiological Estimates in Pompe Disease: Findings from a Scoping Review. Orphanet J. Rare Dis..

[6] Toscano A., Schoser B. (2013). Enzyme Replacement Therapy in Late-Onset Pompe Disease: A Systematic Literature Review. J. Neurol..

[7] Angelini C., Semplicini C., Ravaglia S., Bembi B., Servidei S., Pegoraro E., Moggio M., Filosto M., Sette E., Crescimanno G. (2012). Observational Clinical Study in Juvenile-Adult Glycogenosis Type 2 Patients Undergoing Enzyme Replacement Therapy for up to 4 Years. J. Neurol..

[8] Desai A.K., Walters C.K., Cope H.L., Kazi Z.B., DeArmey S.M., Kishnani P.S. (2018). Enzyme Replacement Therapy with Alglucosidase Alfa in Pompe Disease: Clinical Experience with Rate Escalation. Mol. Genet. Metab..

[9] Sellier P., Vidal P., Bertin B., Gicquel E., Bertil-Froidevaux E., Georger C., van Wittenberghe L., Miranda A., Daniele N., Richard I. (2024). Muscle-Specific, Liver-Detargeted Adeno-Associated Virus Gene Therapy Rescues Pompe Phenotype in Adult and Neonate Gaa^−/−^ Mice. J. Inherit. Metab. Dis..

[10] Douillard-Guilloux G., Richard E., Batista L., Caillaud C. (2009). Partial Phenotypic Correction and Immune Tolerance Induction to Enzyme Replacement Therapy after Hematopoietic Stem Cell Gene Transfer of Alpha-Glucosidase in Pompe Disease. J. Gene Med..

[11] Korlimarla A., Lim J.-A., Kishnani P.S., Sun B. (2019). An Emerging Phenotype of Central Nervous System Involvement in Pompe Disease: From Bench to Bedside and Beyond. Ann. Transl. Med..

[12] Salabarria S.M., Nair J., Clement N., Smith B.K., Raben N., Fuller D.D., Byrne B.J., Corti M. (2020). Advancements in AAV-Mediated Gene Therapy for Pompe Disease. J. Neuromuscul. Dis..

[13] Dasouki M., Jawdat O., Almadhoun O., Pasnoor M., McVey A.L., Abuzinadah A., Herbelin L., Barohn R.J., Dimachkie M.M. (2014). Pompe Disease: Literature Review and Case Series. Neurol. Clin..

[14] Porcino M., Musumeci O., Usbergo C., Pugliese A., Arena I.G., Rodolico C., Schoser B., Toscano A. (2025). Management of Presymptomatic Juvenile Patients with Late-Onset Pompe Disease (LOPD). Neuromuscul. Disord..

[15] Labella B., Cotti Piccinelli S., Risi B., Caria F., Damioli S., Bertella E., Poli L., Padovani A., Filosto M. (2023). A Comprehensive Update on Late-Onset Pompe Disease. Biomolecules.

[16] Holzwarth J., Minopoli N., Pfrimmer C., Smitka M., Borrel S., Kirschner J., Muschol N., Hartmann H., Hennermann J.B., Neubauer B.A. (2022). Clinical and Genetic Aspects of Juvenile Onset Pompe Disease. Neuropediatrics.

[17] Kroos M., Hoogeveen-Westerveld M., van der Ploeg A., Reuser A.J.J. (2012). The Genotype-Phenotype Correlation in Pompe Disease. Am. J. Med. Genet. C Semin. Med. Genet..

[18] Torri F., Buchignani B., Unluturk Z., Vadi G., Loprieno S., Battini R., Mancuso M., Siciliano G. (2025). The Involvement of Central Nervous System across the Phenotypic Spectrum of Pompe Disease: A Systematic Review. Neuromuscul. Disord..

[19] Kishnani P.S., Steiner R.D., Bali D., Berger K., Byrne B.J., Case L.E., Crowley J.F., Downs S., Howell R.R., Kravitz R.M. (2006). Pompe Disease Diagnosis and Management Guideline. Genet. Med..

[20] Peruzzo P., Pavan E., Dardis A. (2019). Molecular Genetics of Pompe Disease: A Comprehensive Overview. Ann. Transl. Med..

[21] Hermans M.M., De Graaff E., Kroos M.A., Mohkamsing S., Eussen B.J., Joosse M., Willemsen R., Kleijer W.J., Oostra B.A., Reuser A.J. (1994). The Effect of a Single Base Pair Deletion (Delta T525) and a C1634T Missense Mutation (Pro545leu) on the Expression of Lysosomal Alpha-Glucosidase in Patients with Glycogen Storage Disease Type II. Hum. Mol. Genet..

[22] Li C., Desai A.K., Gupta P., Dempsey K., Bhambhani V., Hopkin R.J., Ficicioglu C., Tanpaiboon P., Craigen W.J., Rosenberg A.S. (2021). Transforming the Clinical Outcome in CRIM-Negative Infantile Pompe Disease Identified via Newborn Screening: The Benefits of Early Treatment with Enzyme Replacement Therapy and Immune Tolerance Induction. Genet. Med..

[23] Guevara-Campos J., González-Guevara L., Cauli O. (2019). Skeletal Alterations, Developmental Delay and New Mutations in Juvenile-Onset Pompe Disease. Neuromuscul. Disord..

[24] Taverna S., Cammarata G., Colomba P., Sciarrino S., Zizzo C., Francofonte D., Zora M., Scalia S., Brando C., Curto A.L. (2020). Pompe Disease: Pathogenesis, Molecular Genetics and Diagnosis. Aging.

[25] Al-Hassnan Z., Hashmi N.A., Makhseed N., Omran T.B., Al Jasmi F., Teneiji A.A. (2022). Expert Group Consensus on Early Diagnosis and Management of Infantile-Onset Pompe Disease in the Gulf Region. Orphanet J. Rare Dis..

[26] Colburn R., Lapidus D. (2023). An Analysis of Pompe Newborn Screening Data: A New Prevalence at Birth, Insight and Discussion. Front. Pediatr..

[27] Kazi Z.B., Desai A.K., Berrier K.L., Troxler R.B., Wang R.Y., Abdul-Rahman O.A., Tanpaiboon P., Mendelsohn N.J., Herskovitz E., Kronn D. (2017). Sustained Immune Tolerance Induction in Enzyme Replacement Therapy-Treated CRIM-Negative Patients with Infantile Pompe Disease. JCI Insight.

[28] Tarnopolsky M., Katzberg H., Petrof B.J., Sirrs S., Sarnat H.B., Myers K., Dupré N., Dodig D., Genge A., Venance S.L. (2016). Pompe Disease: Diagnosis and Management. Evidence-Based Guidelines from a Canadian Expert Panel. Can. J. Neurol. Sci..

[29] Bali D.S., Goldstein J.L., Banugaria S., Dai J., Mackey J., Rehder C., Kishnani P.S. (2012). Predicting Cross-Reactive Immunological Material (CRIM) Status in Pompe Disease Using GAA Mutations: Lessons Learned from 10 Years of Clinical Laboratory Testing Experience. Am. J. Med. Genet. C Semin. Med. Genet..

[30] Banugaria S.G., Prater S.N., Ng Y.-K., Kobori J.A., Finkel R.S., Ladda R.L., Chen Y.-T., Rosenberg A.S., Kishnani P.S. (2011). The Impact of Antibodies on Clinical Outcomes in Diseases Treated with Therapeutic Protein: Lessons Learned from Infantile Pompe Disease. Genet. Med..

[31] Marques J.S. (2022). The Clinical Management of Pompe Disease: A Pediatric Perspective. Children.

[32] Mendelsohn N.J., Messinger Y.H., Rosenberg A.S., Kishnani P.S. (2009). Elimination of Antibodies to Recombinant Enzyme in Pompe’s Disease. N. Engl. J. Med..

[33] Ratnasingam S., Walker P.A., Tran H., Kaplan Z.S., McFadyen J.D., Tran H., Teh T.-C., Fleming S., Catalano J.V., Chunilal S.D. (2016). Bortezomib-Based Antibody Depletion for Refractory Autoimmune Hematological Diseases. Blood Adv..

[34] Zhao Z., Hua Z., Luo X., Li Y., Yu L., Li M., Lu C., Zhao T., Liu Y. (2022). Application and Pharmacological Mechanism of Methotrexate in Rheumatoid Arthritis. Biomed. Pharmacother..

[35] Lopez-Olivo M.A., Amezaga Urruela M., McGahan L., Pollono E.N., Suarez-Almazor M.E. (2015). Rituximab for Rheumatoid Arthritis. Cochrane Database Syst. Rev..

[36] Thurberg B.L., Lynch Maloney C., Vaccaro C., Afonso K., Tsai A.C.-H., Bossen E., Kishnani P.S., O’Callaghan M. (2006). Characterization of Pre- and Post-Treatment Pathology after Enzyme Replacement Therapy for Pompe Disease. Lab. Investig..

[37] Pena L.D.M., Proia A.D., Kishnani P.S. (2015). Postmortem Findings and Clinical Correlates in Individuals with Infantile-Onset Pompe Disease. JIMD Rep..

[38] Hsu Y.-K., Chien Y.-H., Shinn-Forng Peng S., Hwu W.-L., Lee W.-T., Lee N.-C., Po-Yu Huang E., Weng W.-C. (2023). Evaluating Brain White Matter Hyperintensity, IQ Scores, and Plasma Neurofilament Light Chain Concentration in Early-Treated Patients with Infantile-Onset Pompe Disease. Genet. Med..

[39] van den Dorpel J.J.A., Mackenbach M.J., Dremmen M.H.G., van der Vlugt W.M.C., Rizopoulos D., van Doorn P.A., van der Ploeg A.T., Muetzel R., van der Beek N.A.M.E., van den Hout J.M.P. (2024). Long Term Survival in Patients with Classic Infantile Pompe Disease Reveals a Spectrum with Progressive Brain Abnormalities and Changes in Cognitive Functioning. J. Inherit. Metab. Dis..

[40] Chien Y.-H., Lee N.-C., Peng S.-F., Hwu W.-L. (2006). Brain Development in Infantile-Onset Pompe Disease Treated by Enzyme Replacement Therapy. Pediatr. Res..

[41] DeRuisseau L.R., Fuller D.D., Qiu K., DeRuisseau K.C., Donnelly W.H., Mah C., Reier P.J., Byrne B.J. (2009). Neural Deficits Contribute to Respiratory Insufficiency in Pompe Disease. Proc. Natl. Acad. Sci. USA.

[42] Lee N.-C., Peng W.-H., Tsai L.-K., Lu Y.-H., Wang H.-C., Shih Y.-C., Pung Z.-X., Hu H.-Y., Hwu W.-L., Tseng W.-Y.I. (2020). Ultrastructural and Diffusion Tensor Imaging Studies Reveal Axon Abnormalities in Pompe Disease Mice. Sci. Rep..

[43] Sidman R.L., Taksir T., Fidler J., Zhao M., Dodge J.C., Passini M.A., Raben N., Thurberg B.L., Cheng S.H., Shihabuddin L.S. (2008). Temporal Neuropathologic and Behavioral Phenotype of 6neo/6neo Pompe Disease Mice. J. Neuropathol. Exp. Neurol..

[44] Turner S.M.F., Hoyt A.K., ElMallah M.K., Falk D.J., Byrne B.J., Fuller D.D. (2016). Neuropathology in Respiratory-Related Motoneurons in Young Pompe (Gaa^−/−^) Mice. Respir. Physiol. Neurobiol..

[45] Thirumal Kumar D., Umer Niazullah M., Tasneem S., Judith E., Susmita B., George Priya Doss C., Selvarajan E., Zayed H. (2019). A Computational Method to Characterize the Missense Mutations in the Catalytic Domain of GAA Protein Causing Pompe Disease. J. Cell Biochem..

[46] Huie M.L., Chen A.S., Tsujino S., Shanske S., DiMauro S., Engel A.G., Hirschhorn R. (1994). Aberrant Splicing in Adult Onset Glycogen Storage Disease Type II (GSDII): Molecular Identification of an IVS1 (−13T→G) Mutation in a Majority of Patients and a Novel IVS10 (+1GT→CT) Mutation. Hum. Mol. Genet..

[47] Boerkoel C.F., Exelbert R., Nicastri C., Nichols R.C., Miller F.W., Plotz P.H., Raben N. (1995). Leaky Splicing Mutation in the Acid Maltase Gene Is Associated with Delayed Onset of Glycogenosis Type II. Am. J. Hum. Genet..

[48] Kronn D.F., Day-Salvatore D., Hwu W.-L., Jones S.A., Nakamura K., Okuyama T., Swoboda K.J., Kishnani P.S., Pompe Disease Newborn Screening Working Group (2017). Management of Confirmed Newborn-Screened Patients With Pompe Disease Across the Disease Spectrum. Pediatrics.

[49] Niño M.Y., In ’T Groen S.L.M., Bergsma A.J., Beek N.A.M.E., Kroos M., Hoogeveen-Westerveld M., Ploeg A.T., Pijnappel W.W.M.P. (2019). Extension of the Pompe Mutation Database by Linking Disease-associated Variants to Clinical Severity. Hum. Mutat..

[50] Labrousse P., Chien Y.-H., Pomponio R.J., Keutzer J., Lee N.-C., Akmaev V.R., Scholl T., Hwu W.-L. (2010). Genetic Heterozygosity and Pseudodeficiency in the Pompe Disease Newborn Screening Pilot Program. Mol. Genet. Metab..

[51] Kroos M., Pomponio R.J., van Vliet L., Palmer R.E., Phipps M., Van der Helm R., Halley D., Reuser A. (2008). GAA Database Consortium Update of the Pompe Disease Mutation Database with 107 Sequence Variants and a Format for Severity Rating. Hum. Mutat..

[52] Ponce E., Witte D.P., Hirschhorn R., Huie M.L., Grabowski G.A. (1999). Murine Acid Alpha-Glucosidase: Cell-Specific mRNA Differential Expression during Development and Maturation. Am. J. Pathol..

[53] Wisselaar H.A., Kroos M.A., Hermans M.M., van Beeumen J., Reuser A.J. (1993). Structural and Functional Changes of Lysosomal Acid Alpha-Glucosidase during Intracellular Transport and Maturation. J. Biol. Chem..

[54] Lim J.-A., Li L., Raben N. (2014). Pompe Disease: From Pathophysiology to Therapy and Back Again. Front. Aging Neurosci..

[55] Braulke T., Carette J.E., Palm W. (2024). Lysosomal Enzyme Trafficking: From Molecular Mechanisms to Human Diseases. Trends Cell Biol..

[56] Selvan N., Mehta N., Venkateswaran S., Brignol N., Graziano M., Sheikh M.O., McAnany Y., Hung F., Madrid M., Krampetz R. (2021). Endolysosomal N-Glycan Processing Is Critical to Attain the Most Active Form of the Enzyme Acid Alpha-Glucosidase. J. Biol. Chem..

[57] Xia Q., Huang X., Huang J., Zheng Y., March M.E., Li J., Wei Y. (2021). The Role of Autophagy in Skeletal Muscle Diseases. Front. Physiol..

[58] Bonaldo P., Sandri M. (2013). Cellular and Molecular Mechanisms of Muscle Atrophy. Dis. Model. Mech..

[59] Di Malta C., Cinque L., Settembre C. (2019). Transcriptional Regulation of Autophagy: Mechanisms and Diseases. Front. Cell Dev. Biol..

[60] Johansen T., Lamark T. (2011). Selective Autophagy Mediated by Autophagic Adapter Proteins. Autophagy.

[61] Zhao H., Tang M., Liu M., Chen L. (2018). Glycophagy: An Emerging Target in Pathology. Clin. Chim. Acta.

[62] Chan E.Y.W., Kir S., Tooze S.A. (2007). siRNA Screening of the Kinome Identifies ULK1 as a Multidomain Modulator of Autophagy. J. Biol. Chem..

[63] Do H., Meena N.K., Raben N. (2024). Failure of Autophagy in Pompe Disease. Biomolecules.

[64] Inoki K., Kim J., Guan K.-L. (2012). AMPK and mTOR in Cellular Energy Homeostasis and Drug Targets. Annu. Rev. Pharmacol. Toxicol..

[65] Sanchez A.M.J., Csibi A., Raibon A., Cornille K., Gay S., Bernardi H., Candau R. (2012). AMPK Promotes Skeletal Muscle Autophagy through Activation of Forkhead FoxO3a and Interaction with Ulk1. J. Cell Biochem..

[66] Lim J.-A., Sun B., Puertollano R., Raben N. (2018). Therapeutic Benefit of Autophagy Modulation in Pompe Disease. Mol. Ther..

[67] Lim J.-A., Li L., Shirihai O.S., Trudeau K.M., Puertollano R., Raben N. (2017). Modulation of mTOR Signaling as a Strategy for the Treatment of Pompe Disease. EMBO Mol. Med..

[68] Spampanato C., Feeney E., Li L., Cardone M., Lim J.-A., Annunziata F., Zare H., Polishchuk R., Puertollano R., Parenti G. (2013). Transcription Factor EB (TFEB) Is a New Therapeutic Target for Pompe Disease. EMBO Mol. Med..

[69] Settembre C., Di Malta C., Polito V.A., Garcia Arencibia M., Vetrini F., Erdin S., Erdin S.U., Huynh T., Medina D., Colella P. (2011). TFEB Links Autophagy to Lysosomal Biogenesis. Science.

[70] Gatto F., Rossi B., Tarallo A., Polishchuk E., Polishchuk R., Carrella A., Nusco E., Alvino F.G., Iacobellis F., De Leonibus E. (2017). AAV-Mediated Transcription Factor EB (TFEB) Gene Delivery Ameliorates Muscle Pathology and Function in the Murine Model of Pompe Disease. Sci. Rep..

[71] Lim J.-A., Li L., Kakhlon O., Myerowitz R., Raben N. (2015). Defects in Calcium Homeostasis and Mitochondria Can Be Reversed in Pompe Disease. Autophagy.

[72] Tarallo A., Damiano C., Strollo S., Minopoli N., Indrieri A., Polishchuk E., Zappa F., Nusco E., Fecarotta S., Porto C. (2021). Correction of Oxidative Stress Enhances Enzyme Replacement Therapy in Pompe Disease. EMBO Mol. Med..

[73] Engel A.G. (1970). Acid Maltase Deficiency in Adults: Studies in Four Cases of a Syndrome Which May Mimic Muscular Dystrophy or Other Myopathies. Brain.

[74] Raben N., Danon M., Gilbert A.L., Dwivedi S., Collins B., Thurberg B.L., Mattaliano R.J., Nagaraju K., Plotz P.H. (2003). Enzyme Replacement Therapy in the Mouse Model of Pompe Disease. Mol. Genet. Metab..

[75] Shea L., Raben N. (2009). Autophagy in Skeletal Muscle: Implications for Pompe Disease. Int. J. Clin. Pharmacol. Ther..

[76] Fukuda T., Ahearn M., Roberts A., Mattaliano R.J., Zaal K., Ralston E., Plotz P.H., Raben N. (2006). Autophagy and Mistargeting of Therapeutic Enzyme in Skeletal Muscle in Pompe Disease. Mol. Ther..

[77] Lieberman A.P., Puertollano R., Raben N., Slaugenhaupt S., Walkley S.U., Ballabio A. (2012). Autophagy in Lysosomal Storage Disorders. Autophagy.

[78] Raben N., Takikita S., Pittis M.G., Bembi B., Marie S.K.N., Roberts A., Page L., Kishnani P.S., Schoser B.G.H., Chien Y.-H. (2007). Deconstructing Pompe Disease by Analyzing Single Muscle Fibers: To See a World in a Grain of Sand. Autophagy.

[79] Kishnani P.S., Corzo D., Nicolino M., Byrne B., Mandel H., Hwu W.L., Leslie N., Levine J., Spencer C., McDonald M. (2007). Recombinant Human Acid [Alpha]-Glucosidase: Major Clinical Benefits in Infantile-Onset Pompe Disease. Neurology.

[80] Do H.V., Khanna R., Gotschall R. (2019). Challenges in Treating Pompe Disease: An Industry Perspective. Ann. Transl. Med..

[81] Chien Y.-H., Hwu W.-L., Lee N.-C. (2013). Pompe Disease: Early Diagnosis and Early Treatment Make a Difference. Pediatr. Neonatol..

[82] Niño M.Y., Wijgerde M., de Faria D.O.S., Hoogeveen-Westerveld M., Bergsma A.J., Broeders M., van der Beek N.A.M.E., van den Hout H.J.M., van der Ploeg A.T., Verheijen F.W. (2021). Enzymatic Diagnosis of Pompe Disease: Lessons from 28 Years of Experience. Eur. J. Hum. Genet..

[83] Nunes Campos L., Davila Rivera I., Ibañez Alegre D.M., Del Puerto González F.N., Garrido San Juan M., Fernandez Zelcer F., Borgobello D., Gerk A., Sosa L.F., Miretti M.M. (2024). Navigating Pompe Disease Assessment: A Comprehensive Scoping Review. Cureus.

[84] Herzeg A., Borges B., Lianoglou B.R., Gonzalez-Velez J., Canepa E., Munar D., Young S.P., Bali D., Gelb M.H., Chakraborty P. (2023). Intrauterine Enzyme Replacement Therapies for Lysosomal Storage Disorders: Current Developments and Promising Future Prospects. Prenat. Diagn..

[85] Ren J., Ma Y., Ma M., Ding J., Jiang J., Zheng X., Han X. (2023). Rapid Ultra-Performance Liquid Chromatography-Tandem Mass Spectrometry Method for the Simultaneous Determination of Three Characteristic Urinary Saccharide Metabolites in Patients with Glycogen Storage Diseases (Type Ib and II). J. Chromatogr. B Anal. Technol. Biomed. Life Sci..

[86] Piraud M., Pettazzoni M., de Antonio M., Vianey-Saban C., Froissart R., Chabrol B., Young S., Laforêt P., French Pompe Study Group (2020). Urine Glucose Tetrasaccharide: A Good Biomarker for Glycogenoses Type II and III? A Study of the French Cohort. Mol. Genet. Metab. Rep..

[87] Pushkov A., Chudakova D., Zhanin I., Nikitin S., Basargina E., Kuzenkova L., Andreeva D., Vasil’eva M., Vasichkin S., Gabitova A. (2026). Pompe Disease: A Country-Wide Molecular Screening in a Cohort of 15,068 Study Participants. Front. Mol. Biosci..

[88] Kumamoto S., Katafuchi T., Nakamura K., Endo F., Oda E., Okuyama T., Kroos M.A., Reuser A.J.J., Okumiya T. (2009). High Frequency of Acid Alpha-Glucosidase Pseudodeficiency Complicates Newborn Screening for Glycogen Storage Disease Type II in the Japanese Population. Mol. Genet. Metab..

[89] Wang Z., Okamoto P., Keutzer J. (2014). A New Assay for Fast, Reliable CRIM Status Determination in Infantile-Onset Pompe Disease. Mol. Genet. Metab..

[90] Bali D.S., Goldstein J.L., Rehder C., Kazi Z.B., Berrier K.L., Dai J., Kishnani P.S. (2015). Clinical Laboratory Experience of Blood CRIM Testing in Infantile Pompe Disease. Mol. Genet. Metab. Rep..

[91] Lin S., Nateqi J., Weingartner-Ortner R., Gruarin S., Marling H., Pilgram V., Lagler F.B., Aigner E., Martin A.G. (2023). An Artificial Intelligence-Based Approach for Identifying Rare Disease Patients Using Retrospective Electronic Health Records Applied for Pompe Disease. Front. Neurol..

[92] Rustamov J., Rustamov Z., Mohamad M.S., Zaki N., Al Tenaiji A., Al Harbi M., Al Jasmi F. (2024). An Expert Rule-Based Approach for Identifying Infantile-Onset Pompe Disease Patients Using Retrospective Electronic Health Records. Sci. Rep..

[93] Hamed A., Curran C., Gwaltney C., DasMahapatra P. (2019). Mobility Assessment Using Wearable Technology in Patients with Late-Onset Pompe Disease. NPJ Digit. Med..

[94] Ricci G., Baldanzi S., Seidita F., Proietti C., Carlini F., Peviani S., Antonini G., Vianello A., Siciliano G., Italian GSD II Group (2018). A Mobile App for Patients with Pompe Disease and Its Possible Clinical Applications. Neuromuscul. Disord..

[95] Moschetti M., Venezia M., Giacomarra M., Marsana E.M., Zizzo C., Duro G., D’Errico A., Colomba P., Duro G. (2025). Highlights of Precision Medicine, Genetics, Epigenetics and Artificial Intelligence in Pompe Disease. Int. J. Mol. Sci..

[96] Kan S.-H., Huang J.Y., Harb J., Rha A., Dalton N.D., Christensen C., Chan Y., Davis-Turak J., Neumann J., Wang R.Y. (2022). CRISPR-Mediated Generation and Characterization of a Gaa Homozygous c.1935C>A (p.D645E) Pompe Disease Knock-in Mouse Model Recapitulating Human Infantile Onset-Pompe Disease. Sci. Rep..

[97] Huang H.-P., Chen P.-H., Hwu W.-L., Chuang C.-Y., Chien Y.-H., Stone L., Chien C.-L., Li L.-T., Chiang S.-C., Chen H.-F. (2011). Human Pompe Disease-Induced Pluripotent Stem Cells for Pathogenesis Modeling, Drug Testing and Disease Marker Identification. Hum. Mol. Genet..

[98] Takikita S., Myerowitz R., Zaal K., Raben N., Plotz P.H. (2009). Murine Muscle Cell Models for Pompe Disease and Their Use in Studying Therapeutic Approaches. Mol. Genet. Metab..

[99] Aguilar-González A., González-Correa J.E., Barriocanal-Casado E., Ramos-Hernández I., Lerma-Juárez M.A., Greco S., Rodríguez-Sevilla J.J., Molina-Estévez F.J., Montalvo-Romeral V., Ronzitti G. (2022). Isogenic GAA-KO Murine Muscle Cell Lines Mimicking Severe Pompe Mutations as Preclinical Models for the Screening of Potential Gene Therapy Strategies. Int. J. Mol. Sci..

[100] Meinke P., Limmer S., Hintze S., Schoser B. (2019). Assessing Metabolic Profiles in Human Myoblasts from Patients with Late-Onset Pompe Disease. Ann. Transl. Med..

[101] Jat P.S., Noble M.D., Ataliotis P., Tanaka Y., Yannoutsos N., Larsen L., Kioussis D. (1991). Direct Derivation of Conditionally Immortal Cell Lines from an H-2Kb-tsA58 Transgenic Mouse. Proc. Natl. Acad. Sci. USA.

[102] Huang W., Zhang Y., Zhou R. (2022). Induced Pluripotent Stem Cell for Modeling Pompe Disease. Front. Cardiovasc. Med..

[103] Yoshida T., Awaya T., Jonouchi T., Kimura R., Kimura S., Era T., Heike T., Sakurai H. (2017). A Skeletal Muscle Model of Infantile-Onset Pompe Disease with Patient-Specific iPS Cells. Sci. Rep..

[104] Yoshida T., Jonouchi T., Osafune K., Takita J., Sakurai H. (2019). A Liver Model of Infantile-Onset Pompe Disease Using Patient-Specific Induced Pluripotent Stem Cells. Front. Cell Dev. Biol..

[105] Raval K.K., Tao R., White B.E., De Lange W.J., Koonce C.H., Yu J., Kishnani P.S., Thomson J.A., Mosher D.F., Ralphe J.C. (2015). Pompe Disease Results in a Golgi-Based Glycosylation Deficit in Human Induced Pluripotent Stem Cell-Derived Cardiomyocytes. J. Biol. Chem..

[106] Cheng Y.-S., Yang S., Hong J., Li R., Beers J., Zou J., Huang W., Zheng W. (2020). Modeling CNS Involvement in Pompe Disease Using Neural Stem Cells Generated from Patient-Derived Induced Pluripotent Stem Cells. Cells.

[107] Huang H.-P., Chiang W., Stone L., Kang C.-K., Chuang C.-Y., Kuo H.-C. (2019). Using Human Pompe Disease-Induced Pluripotent Stem Cell-Derived Neural Cells to Identify Compounds with Therapeutic Potential. Hum. Mol. Genet..

[108] Wang J., Zhou C.J., Khodabukus A., Tran S., Han S.-O., Carlson A.L., Madden L., Kishnani P.S., Koeberl D.D., Bursac N. (2021). Three-Dimensional Tissue-Engineered Human Skeletal Muscle Model of Pompe Disease. Commun. Biol..

[109] Geel T.M., McLaughlin P.M.J., de Leij L.F.M.H., Ruiters M.H.J., Niezen-Koning K.E. (2007). Pompe Disease: Current State of Treatment Modalities and Animal Models. Mol. Genet. Metab..

[110] Seppälä E.H., Reuser A.J.J., Lohi H. (2013). A Nonsense Mutation in the Acid α-Glucosidase Gene Causes Pompe Disease in Finnish and Swedish Lapphunds. PLoS ONE.

[111] Byrne B., Pope M., Gentry M., Sun R., Gurda B., Trivedi P., Cloutier D., Saba S., Fuller D., Coleman K. (2024). 656P AAV Gene Therapy in the Pompe Canine Model Demonstrates Correction of the Muscle and CNS Disorder with Immune Tolerance to Human GAA. Neuromuscul. Disord..

[112] Bijvoet A.G., van de Kamp E.H., Kroos M.A., Ding J.H., Yang B.Z., Visser P., Bakker C.E., Verbeet M.P., Oostra B.A., Reuser A.J. (1998). Generalized Glycogen Storage and Cardiomegaly in a Knockout Mouse Model of Pompe Disease. Hum. Mol. Genet..

[113] Raben N., Nagaraju K., Lee E., Kessler P., Byrne B., Lee L., LaMarca M., King C., Ward J., Sauer B. (1998). Targeted Disruption of the Acid Alpha-Glucosidase Gene in Mice Causes an Illness with Critical Features of Both Infantile and Adult Human Glycogen Storage Disease Type II. J. Biol. Chem..

[114] Meena N.K., Ng Y., Randazzo D., Weigert R., Puertollano R., Raben N. (2024). Intravital Imaging of Muscle Damage and Response to Therapy in a Model of Pompe Disease. Clin. Transl. Med..

[115] Bragato C., Carra S., Blasevich F., Salerno F., Brix A., Bassi A., Beltrame M., Cotelli F., Maggi L., Mantegazza R. (2020). Glycogen Storage in a Zebrafish Pompe Disease Model Is Reduced by 3-BrPA Treatment. Biochim. Biophys. Acta Mol. Basis Dis..

[116] Iacono R., Paragliola F.M.P., Strazzulli A., Moracci M. (2025). A Stable GH31 α-Glucosidase as a Model System for the Study of Mutations Leading to Human Glycogen Storage Disease Type II. J. Enzyme Inhib. Med. Chem..

[117] Batzoglou S., Pachter L., Mesirov J.P., Berger B., Lander E.S. (2000). Human and Mouse Gene Structure: Comparative Analysis and Application to Exon Prediction. Genome Res..

[118] Neufeld E.F. (2011). From Serendipity to Therapy. Annu. Rev. Biochem..

[119] Dahms N.M., Lobel P., Kornfeld S. (1989). Mannose 6-Phosphate Receptors and Lysosomal Enzyme Targeting. J. Biol. Chem..

[120] Van der Ploeg A.T., Loonen M.C., Bolhuis P.A., Busch H.M., Reuser A.J., Galjaard H. (1988). Receptor-Mediated Uptake of Acid Alpha-Glucosidase Corrects Lysosomal Glycogen Storage in Cultured Skeletal Muscle. Pediatr. Res..

[121] van der Ploeg A.T., Reuser A.J.J. (2008). Pompe’s Disease. Lancet.

[122] Kikuchi T., Yang H.W., Pennybacker M., Ichihara N., Mizutani M., Van Hove J.L., Chen Y.T. (1998). Clinical and Metabolic Correction of Pompe Disease by Enzyme Therapy in Acid Maltase-Deficient Quail. J. Clin. Investig..

[123] Bijvoet A.G., Kroos M.A., Pieper F.R., Van der Vliet M., De Boer H.A., Van der Ploeg A.T., Verbeet M.P., Reuser A.J. (1998). Recombinant Human Acid Alpha-Glucosidase: High Level Production in Mouse Milk, Biochemical Characteristics, Correction of Enzyme Deficiency in GSDII KO Mice. Hum. Mol. Genet..

[124] Van Hove J.L., Yang H.W., Wu J.Y., Brady R.O., Chen Y.T. (1996). High-Level Production of Recombinant Human Lysosomal Acid Alpha-Glucosidase in Chinese Hamster Ovary Cells Which Targets to Heart Muscle and Corrects Glycogen Accumulation in Fibroblasts from Patients with Pompe Disease. Proc. Natl. Acad. Sci. USA.

[125] Van den Hout J.M.P., Kamphoven J.H.J., Winkel L.P.F., Arts W.F.M., De Klerk J.B.C., Loonen M.C.B., Vulto A.G., Cromme-Dijkhuis A., Weisglas-Kuperus N., Hop W. (2004). Long-Term Intravenous Treatment of Pompe Disease with Recombinant Human Alpha-Glucosidase from Milk. Pediatrics.

[126] Klinge L., Straub V., Neudorf U., Schaper J., Bosbach T., Görlinger K., Wallot M., Richards S., Voit T. (2005). Safety and Efficacy of Recombinant Acid Alpha-Glucosidase (rhGAA) in Patients with Classical Infantile Pompe Disease: Results of a Phase II Clinical Trial. Neuromuscul. Disord..

[127] Amalfitano A., Bengur A.R., Morse R.P., Majure J.M., Case L.E., Veerling D.L., Mackey J., Kishnani P., Smith W., McVie-Wylie A. (2001). Recombinant Human Acid Alpha-Glucosidase Enzyme Therapy for Infantile Glycogen Storage Disease Type II: Results of a Phase I/II Clinical Trial. Genet. Med..

[128] Risi B., Caria F., Bertella E., Giovanelli G., Gatti S., Poli L., Gazzina S., Leggio U., Bozzoni V., Volonghi I. (2025). Management of Pompe Disease alongside and beyond ERT: A Narrative Review. Acta Myol..

[129] Parini R., De Lorenzo P., Dardis A., Burlina A., Cassio A., Cavarzere P., Concolino D., Della Casa R., Deodato F., Donati M.A. (2018). Long Term Clinical History of an Italian Cohort of Infantile Onset Pompe Disease Treated with Enzyme Replacement Therapy. Orphanet J. Rare Dis..

[130] Dornelles A.D., Junges A.P.P., Pereira T.V., Krug B.C., Gonçalves C.B.T., Llerena J.C., Kishnani P.S., de Oliveira H.A., Schwartz I.V.D. (2021). A Systematic Review and Meta-Analysis of Enzyme Replacement Therapy in Late-Onset Pompe Disease. J. Clin. Med..

[131] Toscano A., Pollissard L., Msihid J., van der Beek N., Kishnani P.S., Dimachkie M.M., Berger K.I., DasMahapatra P., Thibault N., Hamed A. (2024). Effect of Avalglucosidase Alfa on Disease-Specific and General Patient-Reported Outcomes in Treatment-Naïve Adults with Late-Onset Pompe Disease Compared with Alglucosidase Alfa: Meaningful Change Analyses from the Phase 3 COMET Trial. Mol. Genet. Metab..

[132] Kishnani P.S., Kronn D., Brassier A., Broomfield A., Davison J., Hahn S.H., Kumada S., Labarthe F., Ohki H., Pichard S. (2023). Safety and Efficacy of Avalglucosidase Alfa in Individuals with Infantile-Onset Pompe Disease Enrolled in the Phase 2, Open-Label Mini-COMET Study: The 6-Month Primary Analysis Report. Genet. Med..

[133] Corsini A. (2025). Improving the Treatment of Pompe Disease with Enzyme Replacement Therapy: Current Strategies and Clinical Evidence. Expert. Opin. Pharmacother..

[134] Blair H.A. (2023). Cipaglucosidase Alfa: First Approval. Drugs.

[135] Schoser B., Roberts M., Byrne B.J., Sitaraman S., Jiang H., Laforêt P., Toscano A., Castelli J., Díaz-Manera J., Goldman M. (2021). Safety and Efficacy of Cipaglucosidase Alfa plus Miglustat versus Alglucosidase Alfa plus Placebo in Late-Onset Pompe Disease (PROPEL): An International, Randomised, Double-Blind, Parallel-Group, Phase 3 Trial. Lancet Neurol..

[136] Byrne B.J., Geberhiwot T., Barshop B.A., Barohn R., Hughes D., Bratkovic D., Desnuelle C., Laforet P., Mengel E., Roberts M. (2017). A Study on the Safety and Efficacy of Reveglucosidase Alfa in Patients with Late-Onset Pompe Disease. Orphanet J. Rare Dis..

[137] Kishnani P., Lachmann R., Mozaffar T., Walters C., Case L., Appleby M., Libri V., Kak M., Wencel M., Landy H. (2019). Safety and Efficacy of VAL-1221, a Novel Fusion Protein Targeting Cytoplasmic Glycogen, in Patients with Late-Onset Pompe Disease. Mol. Genet. Metab..

[138] Koeberl D.D., Case L.E., Desai A., Smith E.C., Walters C., Han S.-O., Thurberg B.L., Young S.P., Bali D., Kishnani P.S. (2020). Improved Muscle Function in a Phase I/II Clinical Trial of Albuterol in Pompe Disease. Mol. Genet. Metab..

[139] Koeberl D.D., Case L.E., Smith E.C., Walters C., Han S.-O., Li Y., Chen W., Hornik C.P., Huffman K.M., Kraus W.E. (2018). Correction of Biochemical Abnormalities and Improved Muscle Function in a Phase I/II Clinical Trial of Clenbuterol in Pompe Disease. Mol. Ther..

[140] Cohen J.L., Chakraborty P., Fung-Kee-Fung K., Schwab M.E., Bali D., Young S.P., Gelb M.H., Khaledi H., DiBattista A., Smallshaw S. (2022). In Utero Enzyme-Replacement Therapy for Infantile-Onset Pompe’s Disease. N. Engl. J. Med..

[141] Landis J.L., Hyland H., Kindel S.J., Punnoose A., Geddes G.C. (2018). Pompe Disease Treatment with Twice a Week High Dose Alglucoside Alfa in a Patient with Severe Dilated Cardiomyopathy. Mol. Genet. Metab. Rep..

[142] Kishnani P.S., Kronn D., Suwazono S., Broomfield A., Llerena J., Al-Hassnan Z.N., Batista J.L., Wilson K.M., Periquet M., Daba N. (2023). Higher Dose Alglucosidase Alfa Is Associated with Improved Overall Survival in Infantile-Onset Pompe Disease (IOPD): Data from the Pompe Registry. Orphanet J. Rare Dis..

[143] van Gelder C.M., Poelman E., Plug I., Hoogeveen-Westerveld M., van der Beek N.A.M.E., Reuser A.J.J., van der Ploeg A.T. (2016). Effects of a Higher Dose of Alglucosidase Alfa on Ventilator-Free Survival and Motor Outcome in Classic Infantile Pompe Disease: An Open-Label Single-Center Study. J. Inherit. Metab. Dis..

[144] Desai A.K., Smith P.B., Yi J.S., Rosenberg A.S., Burt T.D., Kishnani P.S. (2023). Immunophenotype Associated with High Sustained Antibody Titers against Enzyme Replacement Therapy in Infantile-Onset Pompe Disease. Front. Immunol..

[145] Poelman E., Hoogeveen-Westerveld M., Kroos-de Haan M.A., van den Hout J.M.P., Bronsema K.J., van de Merbel N.C., van der Ploeg A.T., Pijnappel W.W.M.P. (2018). High Sustained Antibody Titers in Patients with Classic Infantile Pompe Disease Following Immunomodulation at Start of Enzyme Replacement Therapy. J. Pediatr..

[146] Desai A.K., Li C., Rosenberg A.S., Kishnani P.S. (2019). Immunological Challenges and Approaches to Immunomodulation in Pompe Disease: A Literature Review. Ann. Transl. Med..

[147] Kenney-Jung D., Korlimarla A., Spiridigliozzi G.A., Wiggins W., Malinzak M., Nichting G., Jung S.-H., Sun A., Wang R.Y., Al Shamsi A. (2024). Severe CNS Involvement in a Subset of Long-Term Treated Children with Infantile-Onset Pompe Disease. Mol. Genet. Metab..

[148] Harlaar L., Hogrel J.-Y., Perniconi B., Kruijshaar M.E., Rizopoulos D., Taouagh N., Canal A., Brusse E., van Doorn P.A., van der Ploeg A.T. (2019). Large Variation in Effects during 10 Years of Enzyme Therapy in Adults with Pompe Disease. Neurology.

[149] Lessard L.E.R., Tard C., Salort-Campana E., Sacconi S., Béhin A., Bassez G., Orlikowski D., Merle P., Nollet S., Gallay L. (2023). Hypersensitivity Infusion-Associated Reactions Induced by Enzyme Replacement Therapy in a Cohort of Patients with Late-Onset Pompe Disease: An Experience from the French Pompe Registry. Mol. Genet. Metab..

[150] Gutschmidt K., Musumeci O., Díaz-Manera J., Chien Y.-H., Knop K.C., Wenninger S., Montagnese F., Pugliese A., Tavilla G., Alonso-Pérez J. (2021). STIG Study: Real-World Data of Long-Term Outcomes of Adults with Pompe Disease under Enzyme Replacement Therapy with Alglucosidase Alfa. J. Neurol..

[151] Pillai N.R., Solomon F., Steiner R.D., Xie B., Haselkorn T., Young C., Rozario N., Walzer M., Schoser B. (2025). A Retrospective Cohort Study Describing the Disease Burden in Patients with Pompe Disease Treated with Enzyme Replacement Therapy in the United States. J. Neuromuscul. Dis..

[152] Contributor B. (2024). Addressing the Lack of Treatment Options for Pompe Disease in South Africa. Rare Dis. Advis..

[153] Dhillon S. (2021). Avalglucosidase Alfa: First Approval. Drugs.

[154] Zhu Y., Jiang J.-L., Gumlaw N.K., Zhang J., Bercury S.D., Ziegler R.J., Lee K., Kudo M., Canfield W.M., Edmunds T. (2009). Glycoengineered Acid Alpha-Glucosidase with Improved Efficacy at Correcting the Metabolic Aberrations and Motor Function Deficits in a Mouse Model of Pompe Disease. Mol. Ther..

[155] Dimachkie M.M., Barohn R.J., Byrne B., Goker-Alpan O., Kishnani P.S., Ladha S., Laforêt P., Mengel K.E., Peña L.D.M., Sacconi S. (2022). Long-Term Safety and Efficacy of Avalglucosidase Alfa in Patients With Late-Onset Pompe Disease. Neurology.

[156] Carter C., Boggs T., Case L.E., Kishnani P. (2024). Real-World Outcomes from a Series of Patients with Late Onset Pompe Disease Who Switched from Alglucosidase Alfa to Avalglucosidase Alfa. Front. Genet..

[157] Diaz-Manera J., Kishnani P.S., Kushlaf H., Ladha S., Mozaffar T., Straub V., Toscano A., van der Ploeg A.T., Berger K.I., Clemens P.R. (2021). Safety and Efficacy of Avalglucosidase Alfa versus Alglucosidase Alfa in Patients with Late-Onset Pompe Disease (COMET): A Phase 3, Randomised, Multicentre Trial. Lancet Neurol..

[158] Tard C., Bouhour F., Michaud M., Beltran S., Fournier M., Demurger F., Lagrange E., Nollet S., Sacconi S., Noury J. (2024). Real-life Effectiveness 1 Year after Switching to Avalglucosidase Alfa in Late-onset Pompe Disease Patients Worsening on Alglucosidase Alfa Therapy: A French Cohort Study. Euro. J. Neurol..

[159] Byrne B.J., Parenti G., Schoser B., van der Ploeg A.T., Do H., Fox B., Goldman M., Johnson F.K., Kang J., Mehta N. (2024). Cipaglucosidase Alfa plus Miglustat: Linking Mechanism of Action to Clinical Outcomes in Late-Onset Pompe Disease. Front. Neurol..

[160] Xu S., Lun Y., Frascella M., Garcia A., Soska R., Nair A., Ponery A.S., Schilling A., Feng J., Tuske S. (2019). Improved Efficacy of a Next-Generation ERT in Murine Pompe Disease. JCI Insight.

[161] Schoser B., Kishnani P.S., Bratkovic D., Byrne B.J., Claeys K.G., Díaz-Manera J., Laforêt P., Roberts M., Toscano A., van der Ploeg A.T. (2024). 104-Week Efficacy and Safety of Cipaglucosidase Alfa plus Miglustat in Adults with Late-Onset Pompe Disease: A Phase III Open-Label Extension Study (ATB200-07). J. Neurol..

[162] Maga J.A., Zhou J., Kambampati R., Peng S., Wang X., Bohnsack R.N., Thomm A., Golata S., Tom P., Dahms N.M. (2013). Glycosylation-Independent Lysosomal Targeting of Acid α-Glucosidase Enhances Muscle Glycogen Clearance in Pompe Mice. J. Biol. Chem..

[163] Kissel J.T., McDermott M.P., Mendell J.R., King W.M., Pandya S., Griggs R.C., Tawil R., FSH-DY Group (2001). Randomized, Double-Blind, Placebo-Controlled Trial of Albuterol in Facioscapulohumeral Dystrophy. Neurology.

[164] George K., Riley R., Zhou S., Allen E., Smith L., Kistanova E., Kinton S., Guo L., Bangari D., Ismail A. (2025). Novel Transferrin Receptor-Mediated Enzyme Replacement Therapy Efficiently Treats Myogenic and Neurogenic Aspects of Pompe Disease in Mice. Mol. Ther. Methods Clin. Dev..

[165] Leon-Astudillo C., Trivedi P.D., Sun R.C., Gentry M.S., Fuller D.D., Byrne B.J., Corti M. (2023). Current Avenues of Gene Therapy in Pompe Disease. Curr. Opin. Neurol..

[166] Roger A.L., Sethi R., Huston M.L., Scarrow E., Bao-Dai J., Lai E., Biswas D.D., El Haddad L., Strickland L.M., Kishnani P.S. (2022). What’s New and What’s next for Gene Therapy in Pompe Disease?. Expert. Opin. Biol. Ther..

[167] Koeberl D.D., Koch R.L., Lim J.-A., Brooks E.D., Arnson B.D., Sun B., Kishnani P.S. (2024). Gene Therapy for Glycogen Storage Diseases. J. Inherit. Metab. Dis..

[168] Mozaffar T., Longo N., Walzer M., Steup A., Coats J., Hayashi C., Diaz-Manera J. Two-Year Safety and Exploratory Efficacy of AT845 Gene Replacement Therapy for Late Onset Pompe Disease: FORTIS, a Phase 1/2 Open-Label Clinical Study. Proceedings of the MDA Clinical & Scientific Conference.

[169] Smith E.C., Hopkins S., Case L.E., Xu M., Walters C., Dearmey S., Han S.-O., Spears T.G., Chichester J.A., Bossen E.H. (2023). Phase I Study of Liver Depot Gene Therapy in Late-Onset Pompe Disease. Mol. Ther..

[170] Smith B.K., Collins S.W., Conlon T.J., Mah C.S., Lawson L.A., Martin A.D., Fuller D.D., Cleaver B.D., Clément N., Phillips D. (2013). Phase I/II Trial of Adeno-Associated Virus-Mediated Alpha-Glucosidase Gene Therapy to the Diaphragm for Chronic Respiratory Failure in Pompe Disease: Initial Safety and Ventilatory Outcomes. Hum. Gene Ther..

[171] Corti M., Liberati C., Smith B.K., Lawson L.A., Tuna I.S., Conlon T.J., Coleman K.E., Islam S., Herzog R.W., Fuller D.D. (2017). Safety of Intradiaphragmatic Delivery of Adeno-Associated Virus-Mediated Alpha-Glucosidase (rAAV1-CMV-hGAA) Gene Therapy in Children Affected by Pompe Disease. Hum. Gene Ther. Clin. Dev..

[172] Ma X., Li J., Wang X., Ma W., Wang J., Gu R., Zhu Z., Wang Y., Du Y., Xu J. (2022). First-in-Human Case Report: AAV9-hGAA Gene Therapy for a Patient with Infantile-Onset Pompe Disease. medRxiv.

[173] Colella P., Mingozzi F. (2019). Gene Therapy for Pompe Disease: The Time Is Now. Hum. Gene Ther..

[174] Eggers M., Vannoy C.H., Huang J., Purushothaman P., Brassard J., Fonck C., Meng H., Prom M.J., Lawlor M.W., Cunningham J. (2022). Muscle-Directed Gene Therapy Corrects Pompe Disease and Uncovers Species-Specific GAA Immunogenicity. EMBO Mol. Med..

[175] Hordeaux J., Buza E.L., Dyer C., Goode T., Mitchell T.W., Richman L., Denton N., Hinderer C., Katz N., Schmid R. (2020). Adeno-Associated Virus-Induced Dorsal Root Ganglion Pathology. Hum. Gene Ther..

[176] Johnson E.W., Sutherland J.J., Meseck E., McElroy C., Chand D.H., Tukov F.F., Hudry E., Penraat K. (2023). Neurofilament Light Chain and Dorsal Root Ganglia Injury after Adeno-Associated Virus 9 Gene Therapy in Nonhuman Primates. Mol. Ther. Methods Clin. Dev..

[177] Xu L. Clinical Development of Gene Therapy Products. https://www.fda.gov/media/167536/download.

[178] George L.A., Sullivan S.K., Giermasz A., Ducore J.M., Teitel J.M., Cuker A., Sullivan L.M., Majumdar S., McGuinn C.E., Galvao A.M. (2016). Spk-9001: Adeno-Associated Virus Mediated Gene Transfer for Hemophilia B Achieves Sustained Mean Factor IX Activity Levels of >30% without Immunosuppression. Blood.

[179] Costa-Verdera H., Collaud F., Riling C.R., Sellier P., Nordin J.M.L., Preston G.M., Cagin U., Fabregue J., Barral S., Moya-Nilges M. (2021). Hepatic Expression of GAA Results in Enhanced Enzyme Bioavailability in Mice and Non-Human Primates. Nat. Commun..

[180] Corti M., Cleaver B., Clément N., Conlon T.J., Faris K.J., Wang G., Benson J., Tarantal A.F., Fuller D., Herzog R.W. (2015). Evaluation of Readministration of a Recombinant Adeno-Associated Virus Vector Expressing Acid Alpha-Glucosidase in Pompe Disease: Preclinical to Clinical Planning. Hum. Gene Ther. Clin. Dev..

[181] Song J., Du G., Chen W., Bao P., Li B., Lu Q., Shi B. (2020). The Advantage of Sirolimus in Amplifying Regulatory B Cells and Regulatory T Cells in Liver Transplant Patients. Eur. J. Pharmacol..

[182] Zhao Q., Peng H., Ma Y., Yuan H., Jiang H. (2025). In Vivo Applications and Toxicities of AAV-Based Gene Therapies in Rare Diseases. Orphanet J. Rare Dis..

[183] Costa-Verdera H., Unzu C., Valeri E., Adriouch S., González Aseguinolaza G., Mingozzi F., Kajaste-Rudnitski A. (2023). Understanding and Tackling Immune Responses to Adeno-Associated Viral Vectors. Hum. Gene Ther..

[184] Tsaregorodtseva T.S., Gubaidullina A.A., Kayumova B.R., Shaimardanova A.A., Issa S.S., Solovyeva V.V., Sufianov A.A., Sufianova G.Z., Rizvanov A.A. (2025). Neutralizing Antibodies: Role in Immune Response and Viral Vector Based Gene Therapy. Int. J. Mol. Sci..

[185] Wright J.F. (2023). Re-Administration of AAV Vectors by Masking with Host Albumin: A Goldilocks Hypothesis. Mol. Ther..

[186] Yabe H. (2022). Allogeneic Hematopoietic Stem Cell Transplantation for Inherited Metabolic Disorders. Int. J. Hematol..

[187] Watson J.G., Gardner-Medwin D., Goldfinch M.E., Pearson A.D. (1986). Bone Marrow Transplantation for Glycogen Storage Disease Type II (Pompé’s Disease). N. Engl. J. Med..

[188] Philippidis A. (2024). Orchard Therapeutics Gains First U.S. Approval for a Metachromatic Leukodystrophy Gene Therapy. Hum. Gene Ther..

[189] Keam S.J. (2021). Elivaldogene Autotemcel: First Approval. Mol. Diagn. Ther..

[190] FDA Approves First Cell-Based Gene Therapy to Treat Adult and Pediatric Patients with Beta-Thalassemia Who Require Regular Blood Transfusions|FDA. https://web.archive.org/web/20220821044703/http://www.fda.gov/news-events/press-announcements/fda-approves-first-cell-based-gene-therapy-treat-adult-and-pediatric-patients-beta-thalassemia-who.

[191] Unnisa Z., Yoon J.K., Schindler J.W., Mason C., van Til N.P. (2022). Gene Therapy Developments for Pompe Disease. Biomedicines.

[192] Loeb A.M., Pattwell S.S., Meshinchi S., Bedalov A., Loeb K.R. (2023). Donor Bone Marrow-Derived Macrophage Engraftment into the Central Nervous System of Patients Following Allogeneic Transplantation. Blood Adv..

[193] van Til N.P., Stok M., Aerts Kaya F.S.F., de Waard M.C., Farahbakhshian E., Visser T.P., Kroos M.A., Jacobs E.H., Willart M.A., van der Wegen P. (2010). Lentiviral Gene Therapy of Murine Hematopoietic Stem Cells Ameliorates the Pompe Disease Phenotype. Blood.

[194] Stok M., de Boer H., Huston M.W., Jacobs E.H., Roovers O., Visser T.P., Jahr H., Duncker D.J., van Deel E.D., Reuser A.J.J. (2020). Lentiviral Hematopoietic Stem Cell Gene Therapy Corrects Murine Pompe Disease. Mol. Ther. Methods Clin. Dev..

[195] Liang Q., Stok M., van Helsdingen Y., van der Velden G., Jacobs E., Duncker D., Reuser A., van der Ploeg A., Vulto A., van Til N.P. (2015). Lentiviral Stem Cell Gene Therapy for Pompe Disease. J. Neuromuscul. Dis..

[196] Liang Q., Catalano F., Vlaar E.C., Pijnenburg J.M., Stok M., van Helsdingen Y., Vulto A.G., van der Ploeg A.T., van Til N.P., Pijnappel W.W.M.P. (2022). IGF2-Tagging of GAA Promotes Full Correction of Murine Pompe Disease at a Clinically Relevant Dosage of Lentiviral Gene Therapy. Mol. Ther. Methods Clin. Dev..

[197] Dogan Y., Barese C.N., Schindler J.W., Yoon J.K., Unnisa Z., Guda S., Jacobs M.E., Oborski C., Maiwald T., Clarke D.L. (2022). Screening Chimeric GAA Variants in Preclinical Study Results in Hematopoietic Stem Cell Gene Therapy Candidate Vectors for Pompe Disease. Mol. Ther. Methods Clin. Dev..

[198] Ellison S., Parker H., Bigger B. (2023). Advances in Therapies for Neurological Lysosomal Storage Disorders. J. Inherit. Metab. Dis..

[199] Ullman J.C., Mellem K.T., Xi Y., Ramanan V., Merritt H., Choy R., Gujral T., Young L.E.A., Blake K., Tep S. (2024). Small-Molecule Inhibition of Glycogen Synthase 1 for the Treatment of Pompe Disease and Other Glycogen Storage Disorders. Sci. Transl. Med..

[200] Savage D.B., Zhai L., Ravikumar B., Choi C.S., Snaar J.E., McGuire A.C., Wou S.-E., Medina-Gomez G., Kim S., Bock C.B. (2008). A Prevalent Variant in PPP1R3A Impairs Glycogen Synthesis and Reduces Muscle Glycogen Content in Humans and Mice. PLoS Med..

[201] Cameron J.M., Levandovskiy V., MacKay N., Utgikar R., Ackerley C., Chiasson D., Halliday W., Raiman J., Robinson B.H. (2009). Identification of a Novel Mutation in GYS1 (Muscle-Specific Glycogen Synthase) Resulting in Sudden Cardiac Death, That Is Diagnosable from Skin Fibroblasts. Mol. Genet. Metab..

[202] Ullman J.C., Linzner D., Tep S., Minga T.D.R., Leeds J. (2024). Muscle Glycogen Reduction in Healthy Adults Treated with MZE001, an Oral Inhibitor of GYS1 and Potential Substrate Reduction Therapy for Pompe Disease.

[203] Siegfried J. (2026). Early Data Show Biomarker Reductions, Good Safety for ABX1100 in Late-Onset Pompe Disease. Rare Dis. Advis..

[204] van der Wal E., Bergsma A.J., Pijnenburg J.M., van der Ploeg A.T., Pijnappel W.W.M.P. (2017). Antisense Oligonucleotides Promote Exon Inclusion and Correct the Common c.-32-13T>G GAA Splicing Variant in Pompe Disease. Mol. Ther. Nucleic Acids.

[205] Weiss L., Carrer M., Shmara A., Cheng C., Yin H., Ta L., Boock V., Fazeli Y., Chang M., Paguio M. (2024). Skeletal Muscle Effects of Antisense Oligonucleotides Targeting Glycogen Synthase 1 in a Mouse Model of Pompe Disease. bioRxiv.

